# Pan-Influenza A Protection by Prime–Boost Vaccination with Cold-Adapted Live-Attenuated Influenza Vaccine in a Mouse Model

**DOI:** 10.3389/fimmu.2018.00116

**Published:** 2018-02-01

**Authors:** Yo Han Jang, Joo Young Kim, Young Ho Byun, Ahyun Son, Jeong-Yoon Lee, Yoon Jae Lee, Jun Chang, Baik Lin Seong

**Affiliations:** ^1^Department of Biotechnology, College of Life Science and Biotechnology, Yonsei University, Seoul, South Korea; ^2^Graduate School of Pharmaceutical Sciences, Ewha Womans University, Seoul, South Korea; ^3^Vaccine Translational Research Center, Yonsei University, Seoul, South Korea

**Keywords:** influenza virus, cold-adapted live-attenuated vaccine, cross-protection, T cell, NK cell, antibody

## Abstract

Influenza virus infections continually pose a major public health threat with seasonal epidemics and sporadic pandemics worldwide. While currently licensed influenza vaccines provide only strain-specific protection, antigenic drift and shift occasionally render the viruses resistant to the host immune responses, which highlight the need for a vaccine that provides broad protection against multiple subtypes. In this study, we suggest a vaccination strategy using cold-adapted, live attenuated influenza vaccines (CAIVs) to provide a broad, potent, and safe cross-protection covering antigenically distinct hemagglutinin (HA) groups 1 and 2 influenza viruses. Using a mouse model, we tested different prime–boost combinations of CAIVs for their ability to induce humoral and T-cell responses, and protective efficacy against H1 and H5 (HA group 1) as well as H3 and H7 (HA group 2) influenza viruses. Notably, even in the absence of antibody-mediated neutralizing activity or HA inhibitory activity *in vitro*, CAIVs provided a potent protection against heterologous and heterosubtypic lethal challenges *in vivo*. Heterologous combination of prime (H1)–boost (H5) vaccine strains showed the most potent cross-protection efficacy. *In vivo* depletion experiments demonstrated not only that T cells and natural killer cells contributed to the cross-protection, but also the involvement of antibody-dependent mechanisms for the cross-protection. Vaccination-induced antibodies did not enhance the infectivity of heterologous viruses, and prime vaccination did not interfere with neutralizing antibody generation by the boost vaccination, allaying vaccine safety concerns associated with heterogeneity between the vaccines and challenge strains. Our data show that CAIV-based strategy can serve as a simple but powerful option for developing a “truly” universal influenza vaccine providing pan-influenza A protection, which has not been achieved yet by other vaccine strategies. The promising results of potency, breadth, and safety demonstrated in the mouse model support further studies in higher animal models for clinical relevance.

## Introduction

Influenza virus is an important respiratory pathogen that causes annual epidemics and occasional pandemics. In each season, the influenza epidemic results in 3–5 million cases of severe illness and 250,000–500,000 deaths worldwide (1). Due to the antigenic diversity and variability of the virus, an influenza vaccine has to be updated almost every year to match circulating strains (2). Currently used influenza vaccines provide only strain-specific protection, primarily by inducing neutralizing antibodies against surface glycoproteins, hemagglutinin (HA) and neuraminidase (NA) of the virus (2). In addition, the occurrence of influenza pandemics were often accompanied by zoonotic spillovers of the surface genes into the human-infecting viruses (3), rendering preexisting vaccines ineffective to newly emerging viruses.

In the last decade, a significant breakthrough has been made in the development of a universal influenza vaccine, triggered by the discovery of rare antibodies specific to the immunogenically subdominant but conserved stalk domain of the influenza HA. To redirect the host immune responses from the HA globular head domain toward this conserved stalk domain, rational vaccine designs were heralded, such as headless HA and chimeric HA vaccines (2, 4). The HA stalk-based approaches have been successful in inducing a broader protection than preexisting influenza vaccines. However, concerns were also raised from a practical standpoint, including the low protective efficacy against different HA group viruses, the necessity of multiple vaccinations to achieve sufficient protective efficacy, and the rare cases of adverse effects such as viral infectivity-enhancing activity of the HA stalk antibodies (5–7). Furthermore, recent studies have isolated mutant influenza viruses each showing resistance to a particular HA stalk-specific antibody (8–10). Meanwhile, a recent study has shown that the chimeric HA strategy can be extended to cold-adapted live-attenuated influenza vaccine (CAIV), in which a prime–boost vaccination with CAIV/split-virus vaccines provided superior protection against the pandemic H1N1 infection compared to two doses of split-virus vaccination in ferret model (11). This study is currently under a clinical trial to examine the feasibility of the strategy in humans (https://clinicaltrials.gov/ct2/show/NCT03300050).

Despite the well-documented cross-protective efficacy, CAIVs remain relatively unexplored in the field of universal influenza vaccine development, most likely due to their inefficiency in inducing systemic antibody responses and the difficulties in genetic engineering to expose the HA stalk to the host immune system. On the other hand, CAIVs have many immunological advantages in terms of cross-protection over the other vaccine platforms, including the delivery of a whole set of antigens, the induction of mucosal IgA antibodies, and T-cell responses, as well as the stimulation of innate immunity (12–15). Perhaps most importantly, it has been widely acknowledged that T-cell immunity, which targets viral proteins that are relatively conserved between different influenza strains, is the key to the cross-protection by natural infection or vaccination (16, 17). Therefore, if all of these factors are combined properly and reinforced by a rational vaccination strategy, a CAIV is expected to serve as a powerful platform for a universal influenza vaccine. To date, many studies have reported the development and evaluation of CAIVs targeting homologous strains and, in a subset of those studies, antigenically closely related strains, addressing the issue of cross-protection (15). However, very few studies have addressed in detail the potential of CAIVs as a reliable platform for a universal influenza vaccine that provides the desired levels of breadth, potency, and safety of cross-protection against diverse influenza subtypes.

In this study, we hypothesized that prime and boost vaccinations with CAIVs would further stimulate the immunological correlates and thus display potent and broad cross-protection efficacy. To test this hypothesis, we designed prime–boost vaccinations with different strains of X-31ca-based CAIVs. Distinct to A/Ann Arbor/6/60ca (H2N2) strain that has been used as a donor strain for currently licensed type A CAIVs, the X-31ca was derived from the parent X-31 virus, a reassortant carrying the HA and NA genes of A/Hong Kong/1/1968 (H3N2) under A/Puerto Rico/8/34 (H1N1) backbone (18). We included two different boosting groups − homosubtypic but heterologous boosting (different strains within H1 subtypes) and heterosubtypic boosting (different strains carrying different HA subtypes) − to evaluate and compare their immune responses and protective efficacy against those of single or homologous boosting group. Mice were vaccinated with various combinations of prime–boost CAIVs, and vaccination-induced antibody and T-cell responses as well as protection efficacy were assessed against antigenically distant HA group 1 (H1 and H5) and group 2 influenza viruses (H3 and H7), which are closely associated with both seasonal epidemics and pandemics worldwide. Additionally, we examined whether our strategy accompanied any adverse effects such as vaccination-associated enhanced respiratory disease (VAERD) or antigenic sin-like phenomenon (19, 20), to address potential safety issues related to heterogeneity between the vaccine and the challenge virus. Promising results of the breadth, potency, and safety of the CAIV-based vaccination strategy support its further development into a reliable universal influenza vaccine technology for clinical use.

## Materials and Methods

### Cell Lines

Madin-Darby canine kidney (MDCK) (ATCC CCL-34) cells were cultured in minimum essential medium (MEM) (HyClone) supplemented with 10% fetal bovine serum (HyClone). Mouse macrophage cell line RAW264.7 (ATCC TIB-71) cells were cultured in Dulbecco’s Modified Eagle Medium (DMEM) (HyClone) supplemented with 10% fetal bovine serum (HyClone).

### Vaccines and Viruses

X-31ca-based CAIVs used in this study are genetic reassortants carrying the surface HA and NA genes of A/Korea/1/09 (H1N1) (GQ131023 and GQ132185), A/New Caledonia/20/99 (H1N1) (CY031336 and CY033624), or A/Indonesia/5/05 (H5N1) (CY116646 and CY116648) virus and the six internal genes from the X-31ca donor backbone strain. The X-31ca was derived from the cold-adaptation of the parent X-31 virus (21, 22), a reassortant carrying the HA and NA genes of A/Hong Kong/1/1968 (H3N2) under A/Puerto Rico/8/34 (H1N1) backbone (18). The GenBank database accession numbers of the six internal genes of the X-31ca are DQ874873 for PB2, DQ874874 for PB1, DQ874875 for PA, DQ874877 for NP, DQ874879 for M, and DQ874880 for NS (22). All the CAIVs have been evaluated for attenuated phenotypes, vaccine efficacy, and safety in animal models in the previous studies (23–25). Challenge viruses included two laboratory strains, PR8 (H1N1) (A/Puerto Rico/8/34) and MA81 (H5N2) (A/aquatic bird/Korea/w81/05), one wild-type virus, Phil82 (H3N2) (A/Philippines/2/82), and one 7:1 genetic reassortant virus, reNet03 (H7N1) (PR8:HA of A/Netherlands/219/03). The mouse lethal dose 50 (MLD_50_) of each of the virus was determined by preliminary study, 5 × 10^3^ PFU for PR8 (H1N1), 1 × 10^4^ PFU for MA81 (H5N2), 5 × 10^4^ PFU for Phil82 (H3N2), and 5 × 10^3^ PFU for reNet03 (H7N1).

### Recombinant Influenza HA Proteins

The HA proteins expressed in insect cells were purchased from Sino Biological (China). The seven different HA proteins were derived from A/California/6/2009 (H1N1), A/Puerto Rico/8/1934 (H1N1), A/Canada/720/2006 (H2N2), A/Indonesia/5/2005 (H5N1), A/Hong Kong/35820/2009 (H9N2), A/Sydney/5/1997 (H3N2), and A/Anhui/1/2013 (H7N9) influenza viruses. We also expressed the HA proteins using bacterial expression system. The HA without transmembrane domain (HAΔTM) (positions 1−531 in H1 numbering) and the stalk region (positions 345−531 in H1 numbering) in the HA2 domain of PR8 (H1N1) and A/Korea/1/09 (H1N1) viruses were produced in *Escherichia coli* expression system, as previously described (26). The transmembrane domain (positions 532−566) was excluded from the expression to enable soluble expression of the HAΔTM protein. The expression plasmid (pLysRS-GE) was transformed into *E. coli* host BL21(DE3)pLysS. After expression, the cell lysates were centrifuged and separated into soluble and pellet fractions and were subjected to SDS-PAGE and visualized by staining with Coomassie brilliant blue R-250. The expressed proteins were purified using nickel affinity chromatography.

### Animal Vaccination and Challenge

All animal studies were carried out in strict accordance with the guidelines of the Ministry of Food and Drug Safety (MFDS) of Korea. The experimental protocols including animal infection with an influenza live virus were reviewed and approved by the Institutional Animal Care and Use Committee (IACUC) and the Institutional Biosafety Committee (IBC) of the Yonsei Laboratory Animal Research Center (YLARC) (permit numbers: IACUC-A-201602-138-02, IACUC-A-201605-203-01, and IACUC-A-201605-205-02). Animal infection with CAIV or live virus was carried out in BSL-2 facility in YLARC. For vaccination, 6-week-old balb/c female mice were primed and boosted with 10^5^ PFU of CAIVs with an interval of two weeks. Challenge was done 35 days after the boosting vaccination, and mice that lost weight greater than 25% were considered non-viable and euthanized. Blood was collected from mice through retro-orbital bleeding under anesthesia, and the lungs and the BALF were taken from sacrificed mice.

### Viral Neutralizing Assays

Microneutralization (MN) assay and HA inhibition (HI) assay were performed to measure neutralizing antibody titers. Before MN assay, sera were pre-treated with a receptor-destroying enzyme at 37°C overnight and then heat-inactivated at 56°C for 30 min. 50 µl of twofold serial dilutions of the sera were incubated with 100 tissue cell infectious dose 50 (TCID_50_) of viruses, and the mixtures were transferred to MDCK cells grown in a 96-well plate for viral infection. Viral infection was determined by the cytopathic effect (CPE), and the MN antibody titer of sera was calculated as the reciprocal of the highest dilution that completely suppressed the CPE. For HI assay, sera were pre-treated with a receptor-destroying enzyme at 37°C overnight and then heat-inactivated at 56°C for 30 min. 25 µl of twofold serial dilutions of the sera were incubated with the same volume of four hemagglutination units of the virus at 37°C for 1 h. A total of 50 µl of 1% chicken red blood cells were added and incubated at 4°C for 1 h. HI antibody titer was calculated as the reciprocal of the highest dilution that completely inhibited hemagglutination.

### ELISA for Antibody Titration

Vaccination-induced antibodies specific to the whole virus or the HA proteins were estimated by ELISA. Ninety-six-well plates were coated with 100 µl of 10^5^ PFU of sucrose-gradient purified viruses or 1 µg/ml of the HA proteins overnight. After blocking with 150 µl of 1% BSA in PBS and washing the plates, the wells were incubated with 100 µl of twofold serial dilutions of sera or BALF for 1 h at RT. After washing the plates, the wells were incubated with 100 µl of HRP-conjugated secondary goat anti-mouse IgG antibodies or IgA antibodies for 1 h at RT. The plates were washed and supplemented with 100 µl of TMB solution and incubated for 30 min at RT in the dark. The reaction was stopped by the addition of 50 µl of 2 N H_2_SO_4_ solution, and OD_450_ was measured on an ELISA reader. Antibody titers were calculated as the endpoint dilution that yielded an OD value greater than the mean + 2 SD of the control group.

### NA Inhibition Assay

To determine the standard virus titer for NA inhibition (NAI) assay, viral NA activity was measured as described elsewhere (27). Ninety-six-well plates were coated overnight with 150 µl of 50 µg/ml fetuin. A total of 100 µl of twofold serial dilutions of influenza viruses dissolved in PBS containing 1% BSA were transferred to the fetuin-coated plates and incubated for 1 h at 37°C. The plates were washed and supplemented with 100 µl of 2.5 µg/ml HRP-conjugated peanut lectin and incubated for 1 h at RT. After washing, 100 µl of TMB solution was added to each well and the reaction was stopped after 5 min by the addition of 50 µl of 2 N H_2_SO_4_. The NA activity was expressed as OD_450_ measured by an ELISA reader. For NAI assay, twofold serial dilutions of sera were mixed with the predetermined titer of the virus with the NA activity corresponding to OD_450_ of 1 and incubated for 1 h at 37°C. The mixtures were then transferred to fetuin-coated plates and subjected to the subsequent procedures described above to measure the NA activity.

### Antibody-Dependent Cell-Mediated Cytotoxicity Assay

Vaccination-induced sera antibodies were examined for their ability to mediate antibody-dependent cell-mediated cytotoxicity (ADCC) activity against virus-infected MDCK cells. We used primary spleen cells isolated from naive mice instead of natural killer (NK) cells as effector cells in ADCC assay, since pure NK cells isolated from the spleen demonstrated very weak ADCC activity in the preliminary study. Confluent MDCK cells grown in 96-well plates were infected with two multiplicities of infection of the influenza virus and incubated in MEM supplemented with 20 µM of Z-VAD-FMK (Promega), pan-caspase inhibitor, and in the absence of trypsin to minimize cytotoxicity from the multicycle viral infection. Eight hours after the infection, the supernatant was removed and 100 µl of sera (1:10 dilution) was added to the MDCK cells and incubated for 1 h at 37°C. After the incubation, 100 µl of mouse spleen cells (10^6^ cells) pre-treated with RBC lysis buffer were added to each well and incubated for 2 h at 37°C. The cytotoxicity of the target MDCK cells was measured in triplicate by lactate dehydrogenase (LDH) release assay.

### NP-Based Virus Infectivity Assay

To examine whether vaccination-induced sera antibodies promote the viral infection of heterologous influenza viruses, NP-based ELISA method (6) was used with minor modifications. Confluent MDCK or RAW264.7 cells grown in a 96-well plate were infected with 100 TCID_50_ of the virus. Twenty-four hours later, the infected cells were washed and fixed by an acetone fixative and 100 µl of anti-influenza A/WSN/33 (H1N1) NP antibodies (26) were added to each well and incubated for 1 h at RT. After the binding of secondary antibodies and washings, OD_490_ was measured by an ELISA reader. The virus infectivity was measured in triplicate and expressed as % of control infection with non-vaccination immune sera.

### Flow Cytometric Analysis of NP-Specific CD8+ T Cells

From vaccinated or control mice, blood was taken at 0 (before challenge), 2, 4, and 6 days after challenge with PR8 (H1N1) or Phil82 (H3N2), and the lungs were taken at 6 days after the challenge from the sacrificed mice. To obtain single-cell suspensions, the lungs were homogenized and filtered through 70-µm cell strainers. After centrifugation, the cells were resuspended in fresh MEM and erythrocytes were removed by RBC lysis buffer. The cells were washed with FACS buffer (0.5% FBS, 0.09% NaN3 in PBS) and were blocked with anti-mouse CD16/CD32 (BD Pharmingen) and 5 µg/ml streptavidin (Invitrogen). The cells were then stained with APC-CD8a mAb (clone 53-6.7; Biolegend), FITC-CD44 mAb (clone IM7; Biolegend), and PE-H-2Kd/NP_147–155_(TYQRTRALV)-tetramer. After staining, the cells were fixed by 2% paraformaldehyde and analyzed using the FACSCalibur flow cytometer (BD Bioscience) and the Flowjo software (TreeStar Inc.).

### *In Vivo* Depletion of T Cells or NK Cells

For the depletion of CD4+ T cells and CD8+ T cells *in vivo*, 200 µg of anti-CD8 mAb (clone 2.43; BioXcell) and anti-CD4 mAb (clone GK1.5; BioXcell) were injected intraperitoneally into mice four times at days 1, 3, 5, and 7 before challenge. Non-depleted control mice were given isotype control rat IgG2b antibodies (clone LTF-2, BioXcell). Blood and lungs were taken from the mice 24 h after the last antibody injection and were subjected to flow cytometry to confirm the depletion. For flow cytometric analysis, we used anti-CD8 mAb (clone 53-6.7; Biolegend) and anti-CD4 mAb (clone RM4-5; Biolegend) directed against different epitope of CD4 and CD8 molecules to that of the depleting antibodies. For the depletion of NK cells, 20 µl of anti-asialo GM1 antiserum (Wako Pure Chemical Industries) were injected intraperitoneally into mice four times at days 1, 3, 5, and 7 before challenge. Non-depleted control mice were given normal rabbit serum (Wako Pure Chemical Industries). The spleens were taken from the mice 24 h after the last antibody injection to confirm the depletion by flow cytometry. For flow cytometric analysis, we used anti-CD3 mAb (clone 17A2; Biolegend) and anti-CD49b mAb (clone DX5; Biolegend).

### Statistical Analysis

All data were expressed as mean ± SD of each cohort, and the difference comparison between two groups was conducted by the unpaired, two-tailed Student’s *t*-test. The difference was considered statistically significant when *P* values were <0.05 (***P < 0.001; **P < 0.01; *P < 0.05).

## Results

### Experimental Design of Vaccination and Challenge in Mice

**Figure 1:**
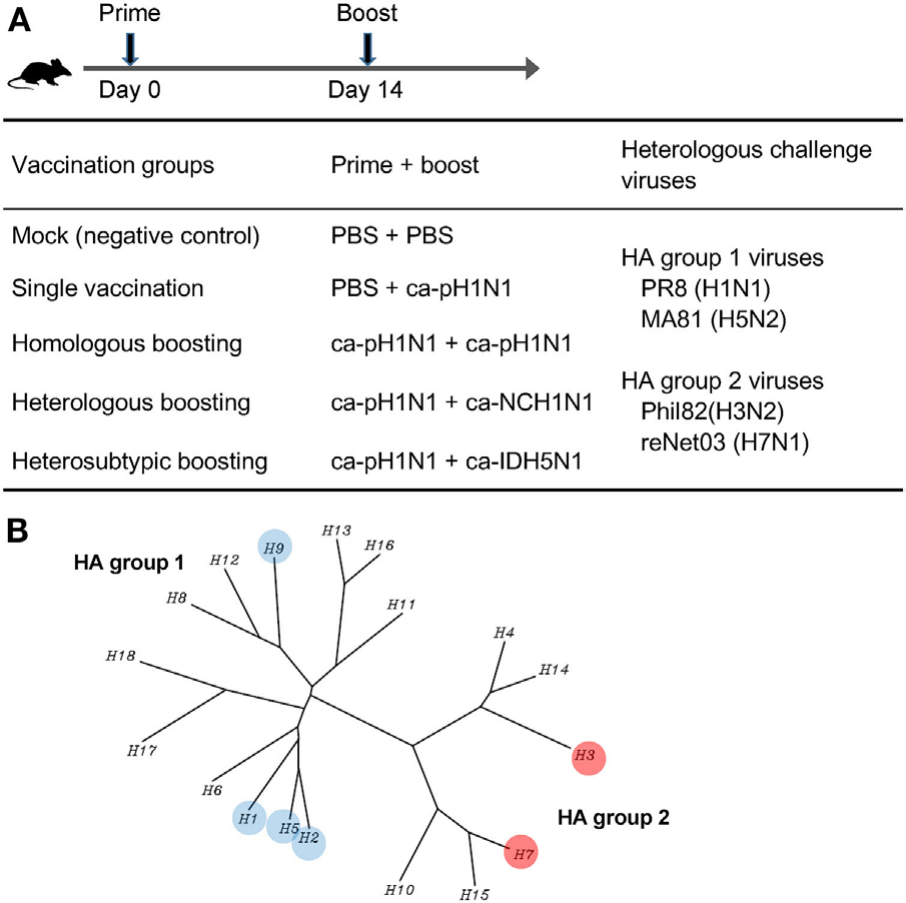
Experimental design of vaccination and challenge in mice. **(A)** Vaccination schedule and design of prime–boost vaccination against heterologous challenge. Four different prime–boost vaccination groups were designed using three different cold-adapted, live attenuated influenza vaccines (CAIVs), ca-pH1N1, ca-NCH1N1, and ca-IDH5N1. Prime and boost CAIVs (10^5^ PFUs of each vaccine) were administered into mice via intranasal route with two weeks interval. A month later, each group was divided into four subgroups (20 subgroups in total) and challenged with 10 mouse lethal dose 50 (MLD_50_) of each of four heterologous influenza viruses. **(B)** Phylogenetic tree of influenza A hemagglutinin (HA) proteins. HA to which binding affinity of vaccination-induced antibodies or protection efficacy *in vivo* tested in this study was highlighted in colored circles.

We previously developed X-31ca as a master strain for CIAVs, by the cold-adaptation of the parent X-31 virus (21, 22). The X-31ca was used as a reliable backbone for CAIVs for the 2009 pandemic A/Korea/1/09 (H1N1) virus (ca-pH1N1), seasonal influenza A/New Caledonia/20/99 (H1N1) virus (ca-NCH1N1), and highly pathogenic H5N1 avian influenza A/Indonesia/5/05 (H5N1) virus (ca-IDH5N1) in subsequent studies (23–25, 28, 29). Using these three CAIVs, four different combinations of prime–boost vaccinations were designed to evaluate and compare induced immune responses and protective efficacy against antigenically distant heterologous influenza viruses in a mouse model. ca-pH1N1 was intranasaly inoculated into the single vaccination group and also used as prime strains in three boosting vaccination groups. Two weeks after the prime vaccination with ca-pH1N1, three different CAIVs were used as boosting strains: ca-pH1N1 for homologous boosting (identical to the prime strain), ca-NCH1N1 for heterologous boosting (different strain of H1N1), and ca-IDH5N1 for heterosubtypic boosting (Figure 1A). Cross-reactive antibody responses elicited by each vaccination were analyzed through various *in vitro* assays as described later. In addition, to evaluate cross-protection *in vivo*, the following four heterologous influenza viruses were used as challenge strains: PR8 (H1N1) and MA81 (H5N2) belonging to the HA group 1 and Phil82 (H3N2) and reNet03 (H7N1) belonging to the HA group 2 (Figure 1B). Thus, while three CAIVs had identical six internal gene segments derived from the X-31ca and the surface genes from different viruses belonging to the HA group 1 (H1 and H5), challenge viruses encompass both HA groups. This experimental design allowed us to assess the potency and breadth of immune responses and protective efficacy against diverse influenza viruses covering both HA groups.

### Cross-Reactive Antibody Responses Induced by Vaccination

**Figure 2:**
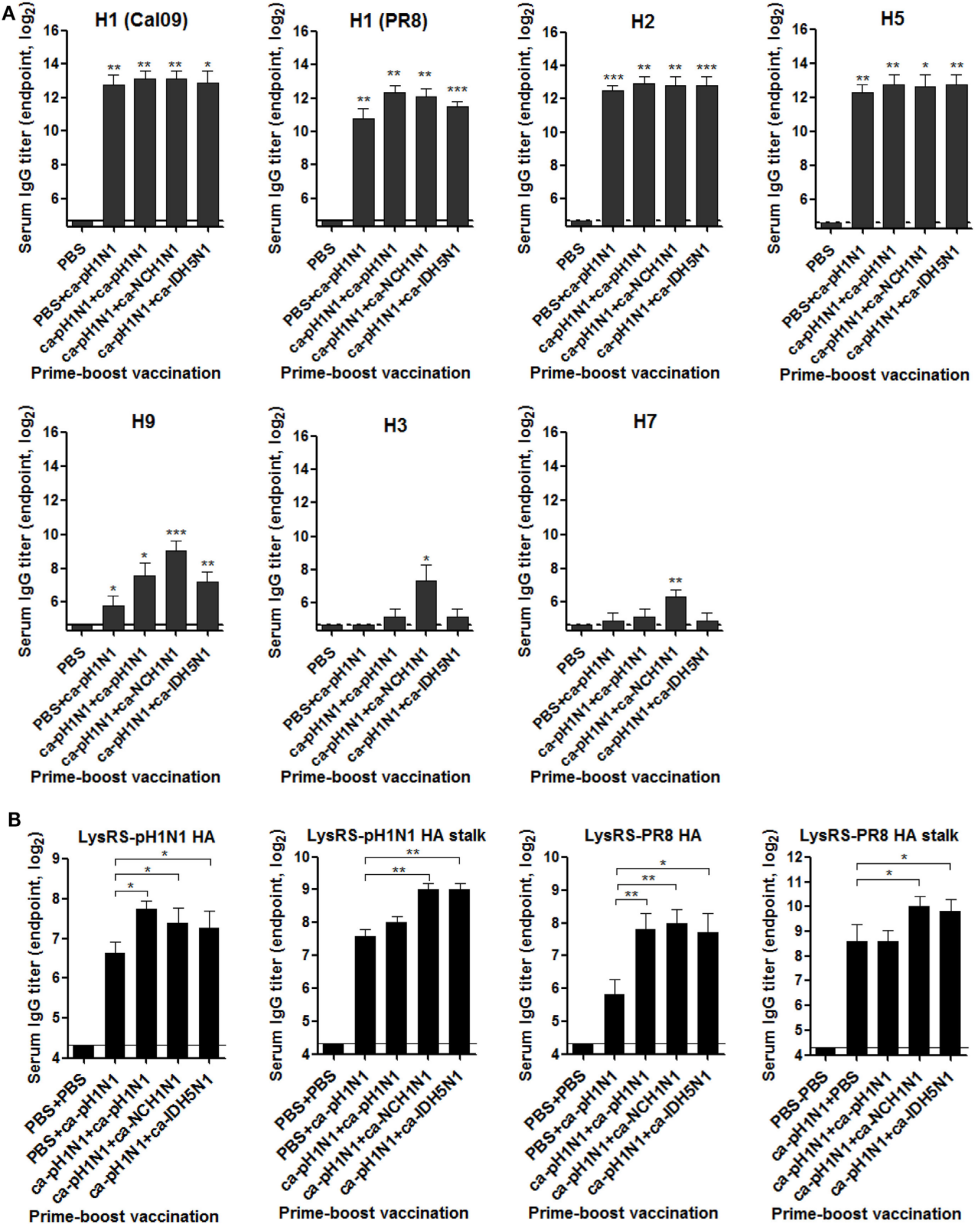
Cross-reactive hemagglutinin (HA)-specific antibody responses elicited by vaccination. **(A)** Breadth of the HA-specific sera IgG antibodies. To examine the breadth of HA-specific sera IgG antibodies induced by vaccination, recombinant HA proteins expressed in insect cells were used as coating antigens in ELISA. The HA proteins tested include five different group 1 HAs from H1N1 (A/California/6/2009), H1N1 (A/Puerto Rico/8/1934), H2N2 (A/Canada/720/2006), H5N1 (A/Indonesia/5/2005), and H9N2 (A/Hong Kong/35820/2009) and two group 2 HAs from H3N2 (A/Sydney/5/1997) and H7N9 (A/Anhui/1/2013) influenza viruses. **(B)** Antibody titers specific to HA full-length or stalk of pH1N1 or PR8 (H1N1) virus. Using the *Escherichia coli*-expressed LysRS-HA fusion proteins as coating antigens, sera IgG antibodies specific to the HA full-length or stalk protein were measured by ELISA. Antibody titers were expressed as the reciprocal serum dilution that yielded OD_450_ greater than the mean + 2 SD (SD) of PBS control group. Data are the mean of each cohort (*N* = 5), and error bars indicate SD. ***P < 0.001; **P < 0.01; *P < 0.05 when comparing the antibody titers between the vaccination group and PBS control group **(A)** or between two different groups **(B)**.

Considering the roles of the HA as a major protective antigen in the protection against influenza viral infection, we first examined whether vaccination-induced sera antibodies bind to the HA proteins of heterologous influenza viruses with ELISA using the insect cell-expressed recombinant HA proteins derived from seven different influenza viruses. All the vaccinations elicited high levels of antibodies cross-reactive to antigenically close group 1 HAs including H1, H2, and H5 (Figure 2A). The robustness of the cross-reactivity of the antibody responses extended to antigenically distant H9 HA, although the antibody titers were significantly reduced as compared to those to the closer HAs. Specific antibody binding was observed even in group 2 HAs (H3 and H7), although noticeable antibody binding seen only in sera from heterologous boosting vaccinations (ca-pH1N1 + ca-NCH1N1) (Figure 2A). Next, we estimated vaccination-induced antibody titers directed to the conserved HA stalk domain. For this purpose, we expressed recombinant HA full-length and stalk proteins derived from A/Korea/1/09 (pH1N1) and PR8 (H1N1) viruses, as soluble proteins fused with the *E. coli* lysyl tRNA synthetase (LysRS) (Figure S1A in Supplementary Material). In ELISA, vaccination-induced antisera strongly bound to the fusion proteins, but not to the LysRS, enabling the measurement of HA-specific antibody titers (Figure S2B in Supplementary Material). The results also suggest that the sequential epitopes present in the HA proteins expressed in *E. coli* can be recognized by specific antibodies. A single vaccination with ca-pH1N1 resulted in a pH1N1-HA-specific antibody titer of 100, and boosting by ca-pH1N1, ca-NCH1N1, and ca-IDH5N1 increased the antibody titers to 210, 160, and 150, respectively (Figure 2B). The most significant increase in the HA-specific antibodies compared to single vaccination was seen in homologous boosting with ca-pH1N1, while heterologous boosting with ca-NCH1N1 and heterosubtypic boosting with ca-IDH5N1 resulted in only modest increases in the HA-specific antibody titers. Vaccinations also resulted in pH1N1-HA stalk-specific antibodies. A single vaccination yielded the HA stalk-specific antibody titer of 192, and each boosting increased the antibody titers to 250−520. In parallel, the cross-reactivity of the antibodies was examined against the PR8 (H1N1) HA protein. A single vaccination induced the PR8 HA antibody titer of 56, and each boosting resulted in a significant increase in the antibody titers ranging 210−260, an approximate fourfold increase of the single vaccination. The PR8 HA stalk-specific antibody levels were very similar between the single vaccination and homologous boosting groups (mean antibody titer of 384 in both). However, heterologous boosting and heterosubtypic boosting resulted in more than a twofold increase in the HA stalk-specific antibody titer compared to that of homologous boosting. The results suggest that heterologous or heterosubtypic boosting could be more effective way of inducing the HA stalk-specific antibodies than the homologous boosting. We also estimated the NA-specific antibodies induced by the vaccination. All the vaccinations generated high levels of antibodies reactive to closely related N1 NA proteins, and the antibody binding reactivity became weak against heterologous N2, N6, N7, N8, and N9 NA proteins (Figure S2 in Supplementary Material). Additionally, vaccination-induced anti-influenza antibodies were measured using purified whole viruses as coating antigens in ELISA. A single vaccination with ca-pH1N1 induced high levels of serum IgG antibodies against not only homologous pH1N1 but also four heterologous influenza viruses, PR8 (H1N1), MA81 (H5N2), Phil82 (H3N2), and reNet03 (H7N1) (Figure S3A in Supplementary Material). Boosting by homologous, heterologous, or heterosubtypic CAIV induced antibody levels similar to, or greater than, that of the single vaccination. IgA antibody levels in the brochoalveolar lavage fluid (BALF) were also estimated against the same set of viruses. Similar to the results of serum IgG antibodies, BALF IgA antibodies showed strong reactivity against all the viruses tested (Figure S3B in Supplementary Material). These results show that the prime–boost vaccination with CAIVs can induce cross-reactive systemic and mucosal antibodies not only against the HA group 1 (H1 and H5) but also against the antigenically distant HA group 2 (H3 and H7) influenza viruses.

### ADCC As a Potential Mechanism of Cross-Protection

**Figure 3:**
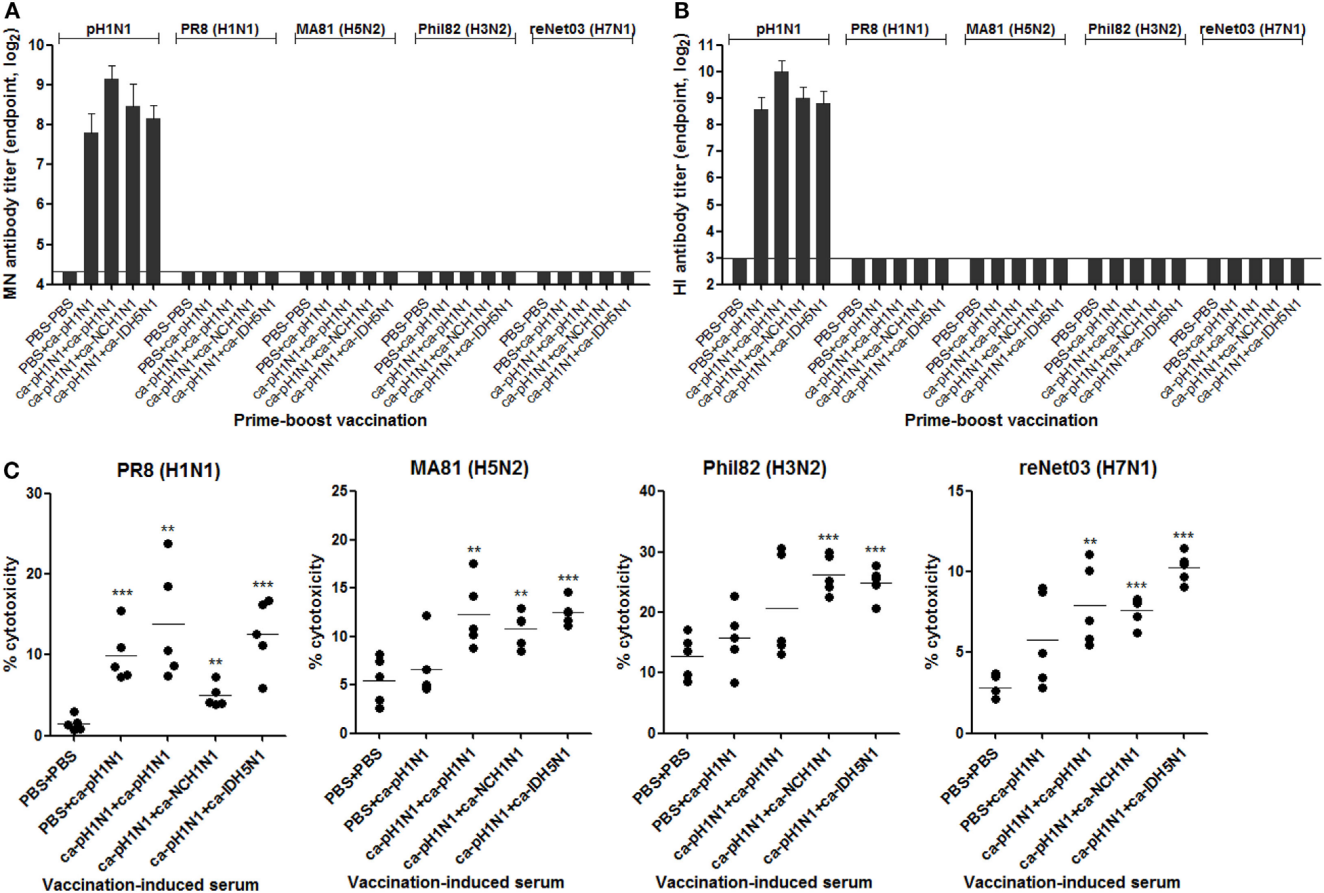
Antibody-dependent mechanism for cross-protection. **(A,B)** Neutralizing activities of sera antibodies. Sera microneutralization (MN) antibody titers **(A)** and HI antibody titers **(B)** against homologous pH1N1 and four heterologous influenza viruses were shown. Detection limits of the MN assay and hemagglutinin inhibition (HI) assay are 20 and 8, respectively. **(C)** antibody-dependent cell-mediated cytotoxicity (ADCC) activity of sera antibodies. Madin-Darby canine kidney (MDCK) cells were pre-infected with two multiplicity of infection of each virus for the expression of viral surface proteins on the cell membrane. ADCC activity on the infected MDCK cells was measured in triplicate by lactate dehydrogenase (LDH) release assay after 2 h of incubation of the cells in the presence of sera (1:10 dilution) and mouse spleen cells as the donor of effector cells. Data are the mean of each cohort (*N* = 5), and error bars indicate SD. ***P < 0.001; **P < 0.01; *P < 0.05 when comparing the cytotoxicity between vaccination group and PBS control group.

To examine whether the cross-reactivity of vaccination-induced antibodies could translate into viral neutralizing activity, we performed various *in vitro* assays with the sera or the mucosal samples. MN assay showed that the sera antibodies effectively inhibited the replication of the homologous pH1N1 virus in MDCK cells. Against the homologous pH1N1, a single vaccination with ca-pH1N1 developed the MN antibody titer of 224, and the homologous, heterologous, and heterosubtypic boosting increased MN antibody titers to 570, 350, and 290, respectively (Figure 3A). However, none of vaccination-induced antibodies yielded detectable levels of MN antibodies against the four heterologous influenza viruses. HI assay with the sera showed the same trend as the MN assay results; sera antibodies could inhibit the hemagglutination activity of the pH1N1 but not the heterologous influenza viruses (Figure 3B). Additionally, we performed an NAI assay with the same set of viruses to examine whether the sea antibodies could block the enzyme activity of the NAs of the heterologous influenza viruses. As expected, homologous boosting yielded the highest level of NAI antibodies against the homologous pH1N1, and heterologous or heterosubtypic boosting resulted in the levels of NAI antibodies similar to that of the single vaccination (Figure S4 in Supplementary Material). However, none of the vaccination-induced antibodies demonstrated NAI activities that were greater than 50% against any of the heterologous viral NAs, with only partial inhibition seen against the heterologous viruses at the least diluted sera.

Recent studies have provided considerable evidence that influenza-specific antibodies have Fc-effector functions, such as ADCC, which target the infected cells for killing by immune cells such as NK cells, monocytes, neutrophils, and macrophages (30–32). We examined whether the vaccination-induced antibodies exert such ADCC activity to MDCK cells infected with the heterologous influenza viruses. All sera from the four vaccination groups resulted in a significant increase in the cytotoxicity of PR8 (H1N1)-infected cells, as compared to the non-vaccination control (Figure 3C). Additionally, sera from the three boosting vaccination groups led to the lysis of MA81 (H5N2)-infected or reNet03 (H7N1)-infected cells. Significant lysis of Phil82 (H3N2)-infected cells was seen by sera from heterologous boosting and heterosubtypic boosting vaccination groups. These results suggest that although the antibodies induced by CAIVs could not neutralize heterologous influenza viruses, they exerted a weak but distinct ADCC activity against the heterologous viruses.

### Cross-Protection against Heterologous Lethal Challenges *In Vivo*

**Figure 4:**
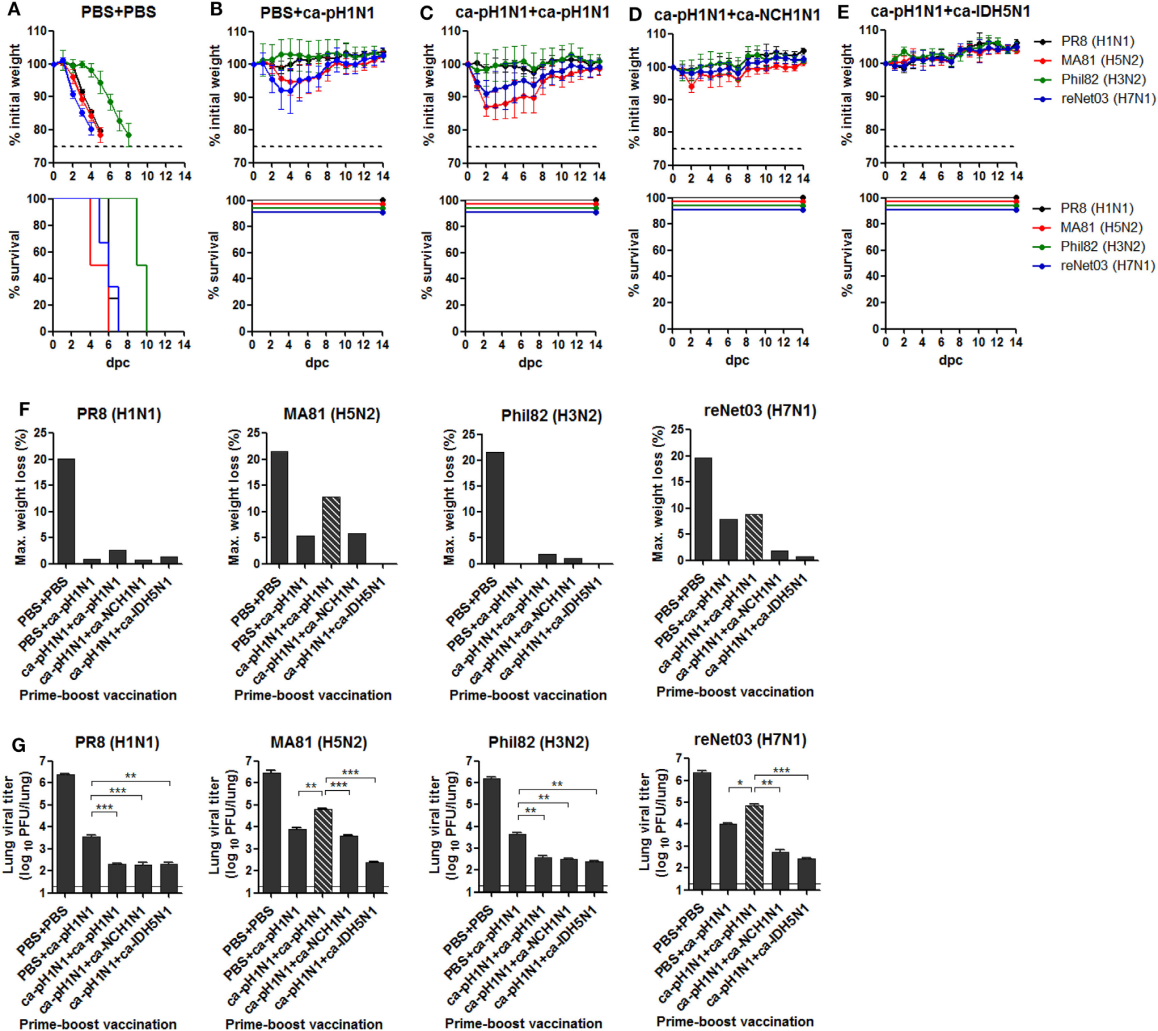
Cross-protection against lethal challenge *in vivo*. **(A−E)** Protection against lethal challenge with heterologous influenza viruses. Control mice that were given PBS **(A)** and vaccinated mice (*N* = 4) **(B−E)** were challenged with 10 mouse lethal dose 50 (MLD_50_) of each of four heterologous influenza viruses and their weight changes (upper) and survival rates (lower) were monitored daily. **(F)** Maximum weight loss of the challenged mice in **(A)** to **(E)**. **(G)** Sterile immunity in the mouse lungs. Separate groups of mice (*N* = 5) vaccinated or given PBS were challenged with 10 MLD_50_ of each heterologous influenza virus. At 6 days post-challenge (dpc), the mice were sacrificed and the lungs were harvested for viral titration by plaque assay. Dashed bars indicate the data of homologous boosting vaccination (ca-pH1N1 + ca-pH1N1) that demonstrated decreased protective efficacy compared to the single vaccination, in terms of maximum weight loss and the lung viral titers. Data are the mean of each cohort, and error bars indicate SD. ***P < 0.001; **P < 0.01; *P < 0.05 when comparing the viral titers between two different groups.

There has been substantial experimental and clinical evidence that CAIVs provide varying degrees of cross-protection against heterologous influenza viruses even without detectable antibody-mediated neutralizing activities (28, 33, 34). To evaluate the breadth and potency of this cross-protection *in vivo*, vaccinated mice were challenged with a lethal dose of each heterologous influenza virus. Non-vaccinated control mice infected with 10 MLD_50_ of PR8 (H1N1), MA81 (H5N2), Phil82 (H3N2), or reNet03 (H7N1) virus showed rapid weight loss and death upon the challenge (Figure 4A). By contrast, a single vaccination with ca-pH1N1 protected the mice from mortality associated with lethal infections, causing mild weight loss of 5−8% upon challenge with MA81 (H5N2) or reNet03 (H7N1), and less than 1% upon challenge with PR8 (H1N1) or Phil82 (H3N2) (Figure 4B). Homologous boosting also resulted in excellent protection against PR8 (H1N1) and Phil82 (H3N2) challenge and resulted in the weight loss of 8.9% following reNet03 (H7N1) challenge, a comparable protective efficacy to that from a single vaccination (Figure 4C). Interestingly, however, MA81 (H5N2) challenge caused weight loss of 12.9% in the homologous boosting group, which was higher than in the single vaccination. Heterologous boosting with ca-NCH1N1 protected the mice from the morbidity associated with the challenges, except for the temporal weight loss of 5.8% at 2 days post-challenge (dpc) with MA81 (H5N2) (Figure 4D). Remarkably, heterosubtypic boosting with ca-IDH5N1 did not resulted in any weight loss after the challenges, showing the most potent cross-protection efficacy among the four vaccination groups (Figure 4E). The virulence represented by weight loss are summarized in Figure 4F. Evidently, challenge with PR8 (H1N1) or Phil82 (H3N2) caused only mild weight loss of less than 3% in all vaccination groups, making it difficult to discriminate the cross-protective efficacy between prime–boost combinations. On the other hand, challenge with MA81 (H5N2) or reNet03 (H7N1) yielded variable weight loss ranging 0−13% depending on the vaccination combination. Homologous boosting demonstrated seemingly lessened protective potency against MA81 (H5N2) or reNet03 (H7N1) as compared to the single vaccination, but the potency was strengthen by heterologous boosting and became complete by heterosubtypic boosting causing weight loss less than 1% upon the challenge. Protective efficacy was further substantiated by estimating challenge virus titers in the lungs of vaccinated mice. All the challenge viruses replicated to greater than 10^6^ PFU in the lungs of the non-vaccinated control group, whereas vaccinations reduced the viral titers to 10^2^−10^4^ PFU (Figure 4G), showing potent sterile immunity. As compared to the single vaccination, each boosting vaccination resulted in an approximate 10-fold reduction of the viral titers of PR8 (H1N1) and Phil82 (H3N2). However, the viral titers of MA81 (H5N2) and reNet03 (H7N1) were higher in the homologous boosting group than the single vaccination group. Again, heterologous boosting and heterosubtypic boosting further decreased the viral titers of MA81 (H5N2) and reNet03 (H7N1). Overall, the protective efficacy evaluated by the weight loss was mirrored in the lung viral titer (Figures 4F,G). The results of protection tests *in vivo* suggest two important points regarding the breadth and potency of our vaccination strategy. First, despite the absence of antibody-mediated viral neutralizing activities, prime–boost vaccination with CAIVs comprising only the HA group 1 strains provided a broad and potent cross-protection covering both HA group 1 and group 2 influenza viruses *in vivo*. Second, the potency of cross-protection against more virulent strains such as H5 and H7 avian influenza viruses could be enhanced through the heterologous or heterosubtypic boosting.

### Contribution of T Cells and NK Cells to Cross-Protection

**Figure 5:**
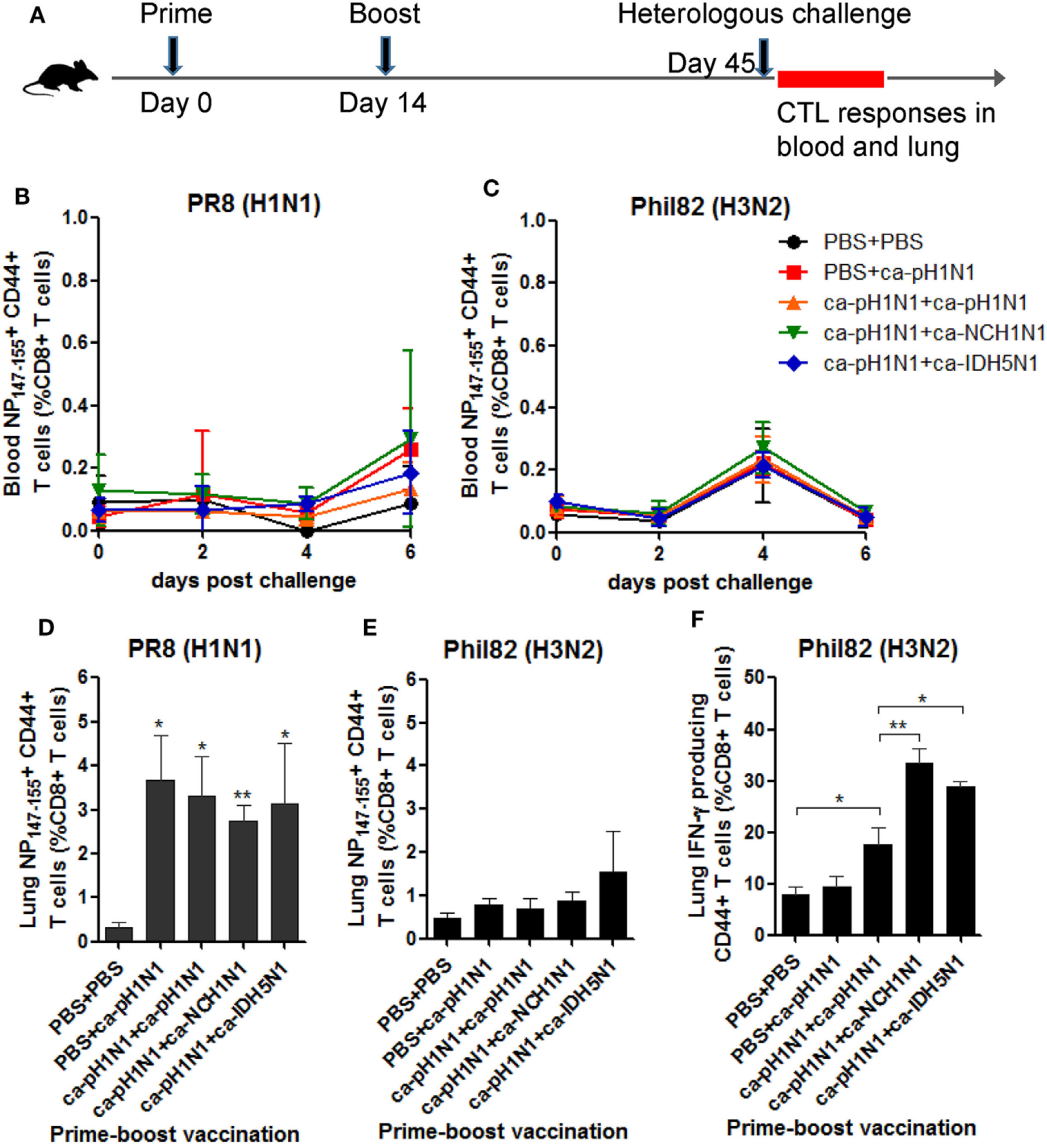
Recall response of CD8+ cytotoxic T lymphocytes (CTLs) upon heterologous challenge. **(A)** Mouse vaccination and challenge for the analysis of CTL responses. A total of 10^5^ PFU of each cold-adapted, live attenuated influenza vaccines (CAIVs) were administered intranasally into naive mice and then challenged with 10 mouse lethal dose 50 (MLD_50_) of PR8 (H1N1) or Phil82 (H3N2). For flow cytometric analysis, blood was taken from the mice at 0, 2, 4, and 6 days post-challenge (dpc), and the mice were sacrificed at 6 dpc to collect the lung cells. **(B−E)** Recall response of NP_147-155_+ CD44+ CD8+ T cells in blood **(B,C)** and the lungs **(D,E)** upon heterologous challenge with PR8 (H1N1) or Phil82 (H3N2) based on flow cytometry data of Figures S5A,B in Supplementary Material, respectively. **(F)** Activation of CD8+ CTLs cells to produce IFN-γ cytokine upon Phil82 (H3N2) challenge based on flow cytometry data of Figure S5C in Supplementary Material. Data are the mean of each cohort (*N* = 3 to 5), and error bars indicate SD. **P < 0.01; *P < 0.05 when comparing the T-cell frequencies between the vaccination group and PBS group **(D,E)** or between two different groups **(F)**.

To further define immunological correlates responsible for the cross-protection, we focused on T cells which have been considered as the key factor for cross-protection. We first examined whether CD8+ cytotoxic T lymphocytes (CTLs) directed to the conserved NP_147−155_ epitope could be recalled in vaccinated mice upon a heterologous challenge. For this purpose, separate groups of mice were primed and boosted with CAIVs and then challenged with PR8 (H1N1) or Phil82 (H3N2), and the specific CTLs in the blood and lungs were analyzed by flow cytometry (Figure 5A). In the peripheral blood, a variable but slightly increased recall of NP_147−155_+ CTLs was observed through six days after the challenge with PR8 (H1N1) or Phil82 (H3N2) (Figures 5B,C). In the lungs, a much more significant increase in the NP_147−155_+ CTLs were observed in the vaccinated groups upon PR8 (H1N1) challenge, in which NP_147−155_+ CTLs accounted for 2.7−3.7% of the whole CD8+ T-cell population, corresponding to a 7.3−9.8-fold increase relative to that of the non-vaccinated control (Figure 5D; Figure S5A in Supplementary Material). Phil82 (H3N2) challenge also recalled the NP_147−155_+ CTLs in the lungs of vaccinated mice, albeit not significantly (Figure 5E; Figure S5B in Supplementary Material). However, IFN-γ producing CTLs were significantly increased upon Phil82 (H3N2) challenge in all three boosting vaccination groups (Figure 5F; Figure S5C in Supplementary Material), suggesting that the boosting vaccination generated CTLs with a specific memory, which then can be re-stimulated to produce IFN-γ cytokine upon the heterologous challenge.

**Figure 6:**
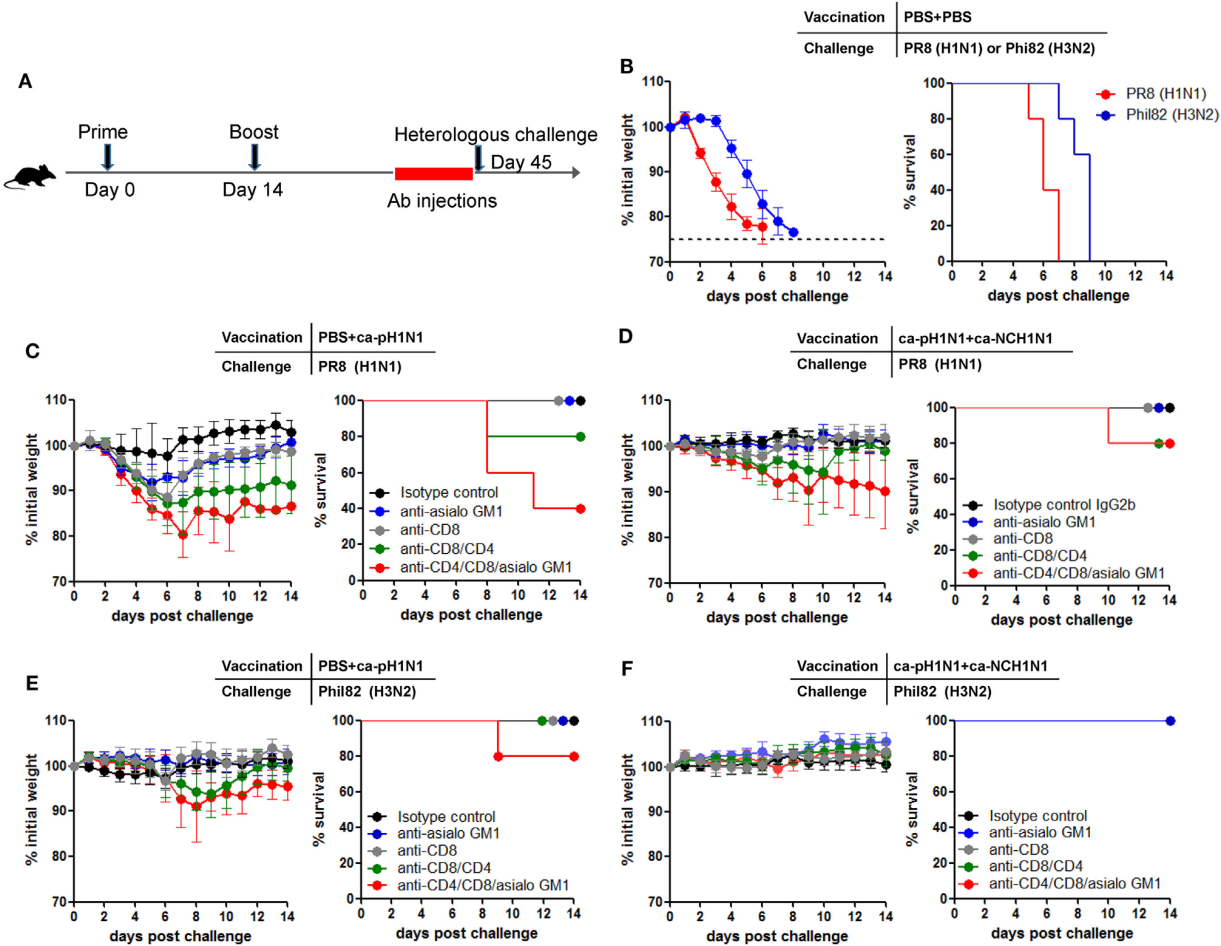
Contribution of CD8+ T cells, CD4+ T cells, and natural killer (NK) cells to cross-protection. **(A)** *In vivo* depletion of CD8+ T cells, CD4+ T cells, or NK cells by injection of antibodies. Depleting antibodies were intraperitoneally injected into mice at 1, 3, 5, and 7 days before challenge. **(B)** Confirmation of the lethality of challenge with 10 mouse lethal dose 50 (MLD_50_) of PR8 (H1N1) or Phil82 (H3N2) in naive normal mice. **(C−F)** Mice (*N* = 5) were vaccinated with PBS + ca-pH1N1 or ca-pH1N1 + ca-NCH1N1, and CD8+ T cells, CD4+ T cells, or NK cells were depleted by the injection of anti-CD8 mAb, anti-CD8 mAb, or anti-asialo GM1 antiserum. The mice were then challenged with 10 MLD_50_ of PR8 (H1N1) or Phil82 (H3N2), and their weight changes and survival rates were monitored daily. Data are the mean of each cohort and error bars indicate SD.

To further determine cell-mediated immunity for cross-protection *in vivo*, a particular subset of the T cells or NK cells were depleted by injecting anti-CD4, anti-CD8, or anti-asialo GM1 antibodies into mice (Figure 6A). Injection of the antibodies to vaccinated mice resulted in the depletion of >98% of CD4+ T cells and CD8+ T cells in the peripheral blood and the lung, and the depletion of 70−80% of NK cells in the spleen (Figures S6A,B in Supplementary Material). First, the lethality of 10 MLD_50_ of PR8 (H1N1) and Phil82 (H3N2) was confirmed in non-vaccinated mice (Figure 6B). Next, separate mice were vaccinated with ca-pH1N1 (single vaccination) or ca-pH1N1 + ca-NCH1N1 (heterologous boosting) and their T cells or NK cells were depleted before challenge with either PR8 (H1N1) or Phil82 (H3N2). Challenge with PR8 (H1N1) caused little morbidity in the single vaccination group that was given isotype control antibodies (Figure 6C). Depletion of CD8+ T cells resulted in a weight loss of ~11% upon the challenge without death. When both CD8+ T cells and CD4+ T cells were depleted, PR8 (H1N1) challenge caused a weight loss of ~13% and death in one of the five mice, and the recovery of the survived mice was significantly delayed (Figure 6C). In a single vaccinated mice, NK cell-depleted group and CD8+ T-cell-depleted group demonstrated similar levels of weight loss upon PR8 (H1N1) challenge, and depletion of both T-cell populations as well as NK cells led to a substantial weight loss of ~20% and the death of three mice (Figure 6C). In heterologous boosting group (ca-pH1N1 + ca-NCH1N1), depletion of both T-cell populations and NK cells resulted in a weight loss of ~10% and the death of one mouse (Figure 6D). Phil82 (H3N2) was also used as a challenge strain. In the single vaccination group, Phil82 (H3N2) challenge caused a weight loss of ~10% and the death of one mouse when CD8+ T cells, CD4+ T cells, and NK cells were depleted (Figure 6E). Heterologous boosting vaccination group did not develop any morbidity upon the Phil82 (H3N2) challenge even in the absence of both T cells and NK cells (Figure 6F). Of note, depletion of CD4+ T cells, CD8+ T cells, and NK cells did not completely abolish cross-protection against the heterologous challenges. These results suggest not only that T cells and NK cells contribute significantly to the cross-protection, but also that there remained other protection mechanisms operating robustly.

### Safety Issues

**Figure 7:**
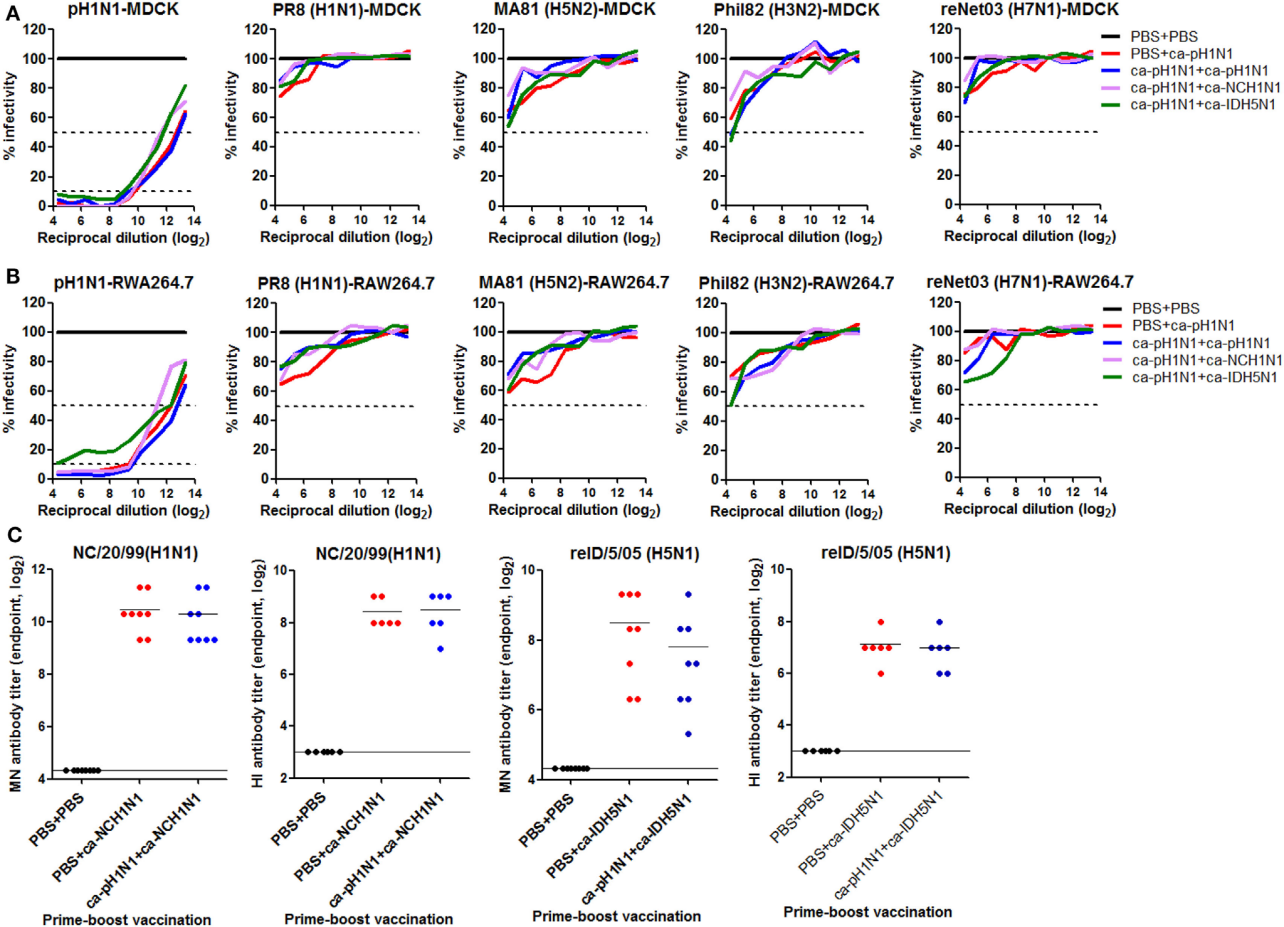
Safety issues related to heterogeneity. **(A,B)** Vaccine-induced sera antibodies do not cause vaccination-associated enhanced respiratory disease (VAERD) by heterologous influenza viruses. Twofold serial dilutions of sera obtained from vaccinated mice (*N* = 5) were incubated with 100 tissue cell infectious dose 50 (TCID_50_) of each virus and the mixtures were absorbed into Madin-Darby canine kidney (MDCK) **(A)** or RAW264.7 **(B)** cells in a 96-well plate. Twenty-four hours later, the viral infectivity of each well was measured by NP-based ELISA protocol. Percent infectivity was calculated based on OD_490_ value compared to that of sera from the non-vaccinated mice. Data are the mean of each cohort. **(C)** Prime vaccination does not interfere with antibody generation by boost vaccination. Mice (*N* = 6 to 8) were vaccinated with PBS + ca-NCH1N1 or PBS + ca-IDH5N1, and sera microneutralization (MN) or hemagglutinin inhibition (HI) antibodies against homologous A/NC/20/1999 or reA/ID/5/2005 virus were estimated and compared to antibody titers generated by vaccination with ca-pH1N1 + ca-NCH1N1 or ca-pH1N1 + ca-IDH5N1.

As described earlier, VAERD has been reported in the cases of influenza virus vaccines, in which non-neutralizing HA stalk antibodies enhanced viral infectivity and aggravated diseases upon heterologous infection (6), raising a concern on the HA stalk-based approaches for cross-protection. Considering that prime–boost vaccinations with CAIVs induced high levels of HA stalk antibodies (Figure 2B), we examined whether the antibodies caused enhanced viral infectivity of the heterologous viruses. Each virus was pre-incubated with the serial dilutions of sera for binding and absorbed into MDCK or RAW264.7 cells, and the viral replication was monitored by NP-based ELISA (6). In both cell lines, vaccination-induced sera antibodies effectively inhibited the replication of the homologous pH1N1 virus, as compared to the non-vaccinated control sera (Figures 7A,B). The sera antibodies showed partial inhibition of the replication of the four heterologous influenza viruses, resulting in the infectivity ranging 50−85%, as compared to the control. However, none of the sera antibodies promoted the viral infectivity of heterologous influenza viruses. The results, along with *in vivo* protection tests described above, suggest that our strategy provides a safe cross-protection against heterologous influenza viruses without causing VAERD by non-neutralizing antibodies. Another potential safety issue of prime–boost vaccination with CAIVs is related to the phenomenon of original antigenic sin. The original antigenic sin theory, first described in 1953 by Thomas Francis (19), refers the phenomenon in which sequential exposure to antigenically different virus strains results in preferential antibody responses to the first strain and impaired immune responses to the second strain. In line with this, recent studies have demonstrated that sequential infections of mice with two different influenza virus strains resulted in almost an exclusive neutralizing antibody response to the first strain and a severely impaired protective immunity to the second strain (20, 35). Thus, we examined if a similar phenomenon occurred in the prime–boost vaccination strategy, in which antigenically heterologous CAIVs were sequentially given to mice. ca-NCH1N1 and ca-IDH5N1 were used as the second vaccine strain in heterologous boosting (ca-pH1N1 + ca-NCH1N1) and heterosubtypic boosting (ca-pH1N1 + ca-IDH5N1) vaccinations, respectively. Neutralizing antibodies to each homologous wild-type virus were estimated by MN and HI assays and were compared to those induced by a single vaccination with ca-NCH1N1 or ca-IDH5N1. A single vaccination with ca-NCH1N1 and heterologous boosting vaccination with ca-pH1N1 + ca-NCH1N1 yielded very similar levels of MN antibody titers (1,330 and 1,270) against the antigenically matched A/NC/20/99 wild-type virus (Figure 7C). Consistently, these two vaccination groups produced similar HI antibody titers (341 and 363) against the same virus. In parallel, a single vaccination with ca-IDH5N1 induced the mean HI and MN antibody titers of 128 and 310, respectively, which were similar to those developed by heterosubtypic boosting vaccination with ca-pH1N1 + ca-IDH5N1 (Figure 7C). The results show that prime vaccination did not prevent the generation of neutralizing antibody responses to the second antigenically heterologous vaccination. Taken together, our data demonstrate that prime–boost vaccination with CAIVs does not accompany adverse effects from heterogeneity not only between the vaccine strains (VAERD) but also between the vaccine and challenge virus (antigenic sin-like phenomenon), supporting the safety of our vaccination strategy.

## Discussion

In this study, we presented prime–boost vaccination with CAIVs as a reliable universal influenza vaccination strategy that conferred a broad protection against diverse influenza viruses. Although the vaccination developed cross-reactive systemic and mucosal antibodies against heterologous influenza viruses, HI and MN assays showed that the antibodies were able to neutralize the homologous virus but not heterologous viruses. Considering that the ELISA using the whole viruses represented the collective results of antibodies to multiple surface proteins including HA, NA, and M2 proteins, further study on the profiles of specific antibodies toward the individual surface proteins would be needed with respect to pan-influenza A protection including HA group 2 viruses. Even without antibody-mediated neutralizing activities, the vaccination provided a complete protection against heterologous lethal challenges and restricted the viral replication in the lungs.

It was unexpected that homologous boosting would demonstrate diminished protective efficacy against MA81 (H5N2) and reNet03 (H7N1) as compared to a single vaccination (Figure 4), considering that homologous boosting generally guarantees improved protection against homologous challenge. It is likely that homologous boosting negatively affected the quality of cross-protection, which could become noticeable when a challenge virus was a highly pathogenic strain such as MA81 (H5N2) or reNet03 (H7N1). This phenomenon merits further investigation into the underlying mechanisms to establish a safe vaccination strategy against virulent strains. Heterologous boosting and heterosubtypic boosting demonstrated a more potent cross-protection against virulent H5 and H7 influenza viruses than the homologous boosting vaccination. Based on the results, we suggest that prime–boost vaccination with CAIVs with genetically and immunologically different HA and NA surface antigens would be a strategy to increase the potency of cross-protection. It is likely that upon a boosting vaccination with heterologous CAIV, potently neutralizing antibodies specific to the immunologically dominant but variable regions are rarely boosted, whereas non- (or less) neutralizing antibodies or CTLs cells directed to the conserved regions are preferentially increased. In support of this assumption, significant increase in the HA stalk antibodies was achieved by heterologous boosting and heterosubtypic boosting but not by homologous boosting (Figure 2B). Furthermore, ADCC activity to antigenically distant H3 and H7 viruses was significant only in the sera from heterologous boosting and heterosubtypic boosting (Figure 3C). Consistent with this, heterologous boosting and heterosubtypic boosting vaccinations demonstrated a higher ability to generate IFN-γ-producing CTLs upon Phil82 (H3N2) challenge than homologous boosting (Figure 5F). PR8 (H1N1) challenge was able to stimulate NP_147−155_+ CTLs although noticeable differences among vaccination groups were not observed (Figure 5D). Parallel experiments with the Phil82 (H3N2) challenge only barely stimulated the NP_147−155_+ CTLs (Figure 5E). These results suggest that the repertoire of activated CTLs can vary according to the challenge virus strain even under the same vaccination conditions. Clearly, further studies are needed on the potential enhancement of CTLs by boost immunization and epitope-specific CTLs responsive to the challenge virus.

The most contrasting feature of our strategy from previously developed HA stalk-based universal vaccines is that the cross-protection elicited by CAIVs did not depend on the neutralizing activities of HA stalk antibodies. Although prime–boost vaccinations with CAIVs induced high levels of HA stalk antibodies, the antibodies exhibited no viral neutralizing activity against the heterologous influenza viruses. HA stalk antibody-mediated inhibition of membrane fusion has been presented as a key mechanism for eliciting a broad protection in vaccine approaches that target the HA stalk (8, 9). It should be noted, however, that most of the HA stalk-based approaches were based on the recombinant HAs carrying the same stalk domains to enable directed boosting toward the conserved region. The current vaccine strategy relied on attenuated viruses carrying phylogenetically distinct HAs carrying relatively conserved and yet different stalk domains. Furthermore, vaccination with CAIVs generally induces a mixture of polyclonal antibodies against the surface antigens including the HA, NA, and M2 as well as the internal proteins, each of which carries a different profile of antigenic epitopes. It remains to be determined whether a heterogeneous profile of antibodies negatively affects the neutralizing activity of the HA stalk antibodies generated in the present study. The induction of polyclonal antibody mixture presents a beneficial effect on cross-protection. There is increasing evidence that ADCC-mediating antibodies directed toward the surface HA, NA, and M2 as well as the internal proteins including NP and M1 were correlated strongly with cross-protection against heterologous influenza viruses (32, 36). Therefore, prime–boost vaccination with CAIVs is likely to induce the whole set of ADCC-mediating antibodies, further increasing the potency of cross-protection. Our data showed that vaccination-induced non-neutralizing antibodies had weak but distinct ADCC activity against heterologous influenza virus-infected MDCK cells (Figure 3C). Consistent with this, depletion of NK cells increased morbidity from PR8 (H1N1) challenge in the single vaccinated mice (Figure 6C). While *in vivo* depletion experiments clearly showed that CD8+ T cells, CD4+ T cells, and NK cells contributed to the cross-protection, the depletion did not completely eliminate the cross-protection elicited by vaccinations, with boosting vaccination providing better protection than the single vaccination (Figure 6). These results suggest that other protective mechanisms are in operation with CAIVs, which can be augmented by boosting vaccination.

To explain possible mechanisms for the cross-protection shown in the present study, careful considerations on various immunological factors potentially induced by a CAIV should be given in a comprehensive manner, since our data did not address all of them individually. One of possible factors is the mucosal IgA antibody-mediated protection in the respiratory tracts. It has been well-known that CAIVs mimic natural infection and thus induce secretory IgA antibodies at the upper and lower respiratory tracts (37–39). Considerable reports have suggested that the secretory IgA antibodies are more cross-protective against influenza virus infections than systemic humoral antibody responses (40–42). Although we could not detect antibody-mediated neutralizing activity in the BALF or nasal turbinates *in vitro* assays, the vaccination resulted in high levels of cross-reactive IgA antibodies (Figure S3B in Supplementary Material), which are likely to play a protective role *in vivo*. CAIVs also develop antibody responses to the M2 external (M2e) domain that is highly conserved across influenza A viruses, and the M2e has long been an attractive target for developing broadly protective universal influenza vaccines (30). In our vaccination strategy, the M2e-specific antibodies also likely play an important role for the cross-protection. Additionally, it has been shown that non-neutralizing antibodies can mediate a number of protective functions, such as complement-dependent cytotoxicity (CDC) and antibody-dependent phagocytosis (ADCP) (43, 44), in addition to ADCC. There is also evidence that innate immune effector cells such as macrophages, monocytes, and neutrophils are capable of inducing ADCC against influenza virus (45–47). In our data, the depletion of CD8+ T cells, CD+ T cells, and NK cells did not completely abolish the protection against heterologous challenges, especially against Phil82 (H3N2) challenge (Figure 6F). It is therefore likely that various types of antibody-dependent protective functions are in operation *in vivo* even after the depletion of CD8+ T cells, CD4+ T cells, and NK cells.

Besides NK cells, the expression of asialo GM1 was also observed in multiple cellular subsets including NKT cells, CD8+ T cells, CD4+ T cells, γδ T cells, macrophages, eosinophils, and basophils (48–53). Thus, it is likely that the injection of the anti-asialo GM1 antibodies affects *in vivo* fates of those cells. Particularly, γδ T cells were shown to contribute to heterosubtypic immunity influenza A virus in the knockout mouse model (12). In our experimental condition, however, the treatment of anti-asialo GM1 antibodies in mice rarely affected the frequencies of the cells expressing CD3 antigen that is expressed in all T-cell populations including NKT cells, CD8+ T cells, CD4+ T cells, and γδ T cells (Figure S6B in Supplementary Material). Still, there remains the possibility that macrophages, eosinophils, and basophils that are important components of cell-mediated innate immune system might be influenced by the anti-asialo GM1 antibodies treatment. These issues underline the need for careful interpretation of phenotypes demonstrated in mice treated with anti-asialo GM1 antibodies.

A truly universal influenza vaccine should be able to guarantee both the potency and the breadth of cross-protection without inducing any adverse effects (5, 54). In this study, we used ca-pH1N1, ca-NCH1N1, and ca-IDH5N1, which are antigenically distinct but close to each another belonging to the HA group 1 (Figure 1B). And yet, prime–boost vaccination with those CAIVs showed such a broad spectrum of protection covering both HA group 1 (H1 and H5) and group 2 (H3 and H7), comprising the viruses of human-infecting and zoonotic potentials. Furthermore, a single vaccination was sufficient to protect mice from death upon the lethal challenge, and boosting substantially improved the potency of protection. Given that these four influenza subtypes present major threats to humans health associated with influenza A viruses, it can safely be concluded that our vaccination strategy confers pan-influenza A protection, which has not been achieved yet by other universal influenza vaccine approaches. It is worth mentioning that, besides the excellent levels of breadth and potency of cross-protection, our vaccination strategy satisfactorily addressed vaccine safety issues, as it did not accompany adverse effects such as VAERD and the antigenic sin phenomenon, both of which sometimes skew the immunogenicity profiles. The promising results of pan-influenza A protection raise an optimistic prospect of developing a pan-influenza universal vaccine that is protective against both influenza A and B viruses simultaneously. Considering the cross-reactive B-cell and T-cell epitopes between influenza A and B viruses (55), it may well be possible to induce such antibodies and T-cell responses through a rational vaccination strategy with CAIVs.

The current study was conducted in a mouse model where the host immune responses to vaccination or infection can be studied in depth (56). It merits further evaluation in higher animal models such as ferrets or swines to better address the clinical relevance and practicality of our strategy. It should also be mentioned that the naive mice had no preexisting immunity to an influenza virus. It has been reported that in humans, preexisting memory B-cell or T-cell immunity affects the recall responses upon the subsequent influenza infection or vaccination (57–59). Our data showed that heterologous prime and boosting vaccinations with an interval of 2 weeks were capable of inducing robust neutralizing antibodies to both strains without any interference between the two vaccines. Additionally, neither heterologous nor heterosubtypic challenge after a month of vaccination accompanied any signs of VAERD. An experimental design with a much longer interval between vaccinations or between vaccination and challenge could provide a greater insight into the relationship between preexisting immunity and protection efficacy against heterologous/heterosubtypic infections.

There remains a possibility that our results of cross-protection were, in part, due to innate immunity such as temporary non-specific immunity or the phenomenon of viral interference by vaccination with a live attenuated virus. Non-specific viral interference between related or non-related viruses has long been observed in humans, and many epidemiological studies have reported clinical cases potentially explained by the phenomenon (60, 61). However, it was reported that non-specific immunity against non-influenza respiratory viruses in children vaccinated with a live-attenuated influenza vaccine (LAIV) was short-lived with the duration of as long as 1−2 weeks (62). Furthermore, many animal studies on cross-protection by LAIVs have presented protection data by performing *in vivo* challenge at 2−4 weeks after the vaccination (11, 17, 33, 63, 64). Considering these observations, our experimental design of the challenge at 5 weeks days after the boosting vaccination cannot be explained by non-specific mechanism. Finally, in our data, *in vivo* protection efficacy against the same challenge strains was significantly different among vaccination regimens (Figure 4), which is unlikely to be mediated by non-specific immunity. In the four vaccinated groups, mice were boosted with the same dose (10^5^ PFU) of CAIV, but their weight loss and the viral replication in the lungs after the challenge suggested the different potency of protection depending on the boosting strain, implying specific adaptive immunity controlling the protection.

There has been an increasing need for a universal influenza vaccine with high levels of breadth, potency, and safety. Harnessing such ideal traits has been proven difficult by using strategies targeting small epitopes or domains alone. Our data demonstrate that prime–boost vaccination with the X-31ca-based CAIV presents as a potentially powerful universal influenza vaccine that provides a broad and potent cross-protection by activating multiple immune arms including antibodies and T cells. Considering that the X-31ca provides the internal backbone of H1N1 strain, it is likely that the X-31ca-based CAIVs demonstrate different profiles of antibody responses and T-cell responses to the internal proteins, as compared to the A/Ann Arbor/6/60ca (H2N2) strain. Whether this difference could bring significant changes in the quality of cross-protection should be addressed by further studies. Whether cross-protection could be extended to influenza B viruses, thus covering all human-infecting viruses, remains ultimate technical challenge for “truly” universal influenza vaccine development.

## Ethics Statement

All animal studies were carried out in strict accordance with the guidelines of the MFDS of Korea. The experimental protocols including animal infection with an influenza live virus were reviewed and approved by the IACUC and the IBC of the YLARC (permit numbers: IACUC-A-201602-138-02, IACUC-A-201605-203-01, and IACUC-A-201605-205-02). Animal infection with CAIV or live virus was carried out in BSL-2 facility in YLARC.

## Author Contributions

YJ and BS designed experiments and wrote the manuscript. YJ, YB, and YL performed animal vaccination and challenge experiments. YJ analyzed antibody responses using ELISA, *in vitro* neutralizing assays, and ADCC assay. AS produced recombinant HA proteins using *E. coli* system. JK and JL performed flow cytometry to measure CTL responses and to confirm the depletion of T cells and NK cells. JC provided technical assistance and contributed reagents. BS supervised all process of experiments and preparation of the manuscript.

## Conflict of Interest Statement

The authors declare that the research was conducted in the absence of any commercial or financial relationships that could be construed as a potential conflict of interest.
