# Supplementary material for: Pan-Influenza A Protection by Prime–Boost Vaccination with Cold-Adapted Live-Attenuated Influenza Vaccine in a Mouse Model
Source: Front Immunol. 2018 Feb 1;9:116. doi: 10.3389/fimmu.2018.00116 (PMC5799225; doi:10.3389/fimmu.2018.00116)
Supplement: Supplementary file 1 [file Presentation_1.PDF]

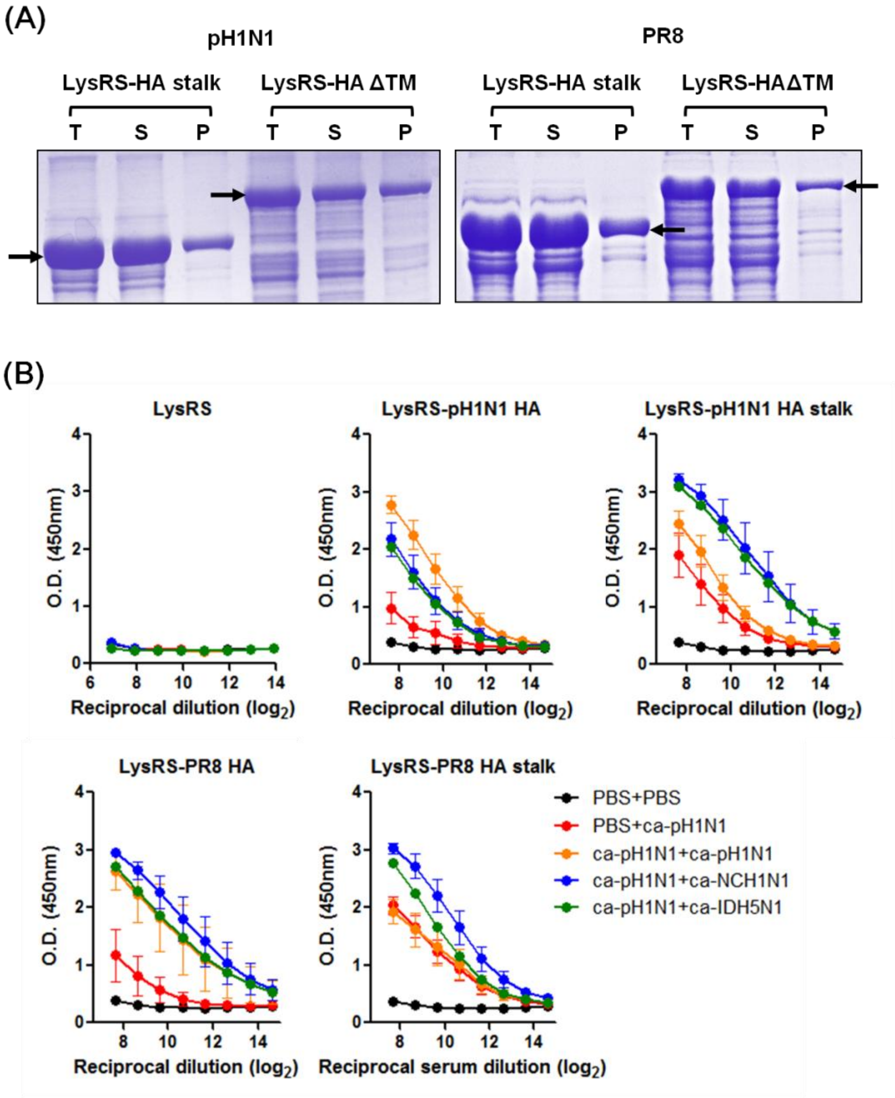

Supplementary Figure 1. Expression of HA proteins in E. coli. (**A**) Bacterial expression of LysRS-fused HA stalk and full-length proteins without transmembrane domain (ΔTM) using E. coli expression system. After induction for protein expression, the bacteria cell lysate was fractionated into total (T), supernatant (S), and pellet (P), and then were subjected to SDS-PAGE to examine the production yield and solubility of the proteins. Arrows indicate the expressed proteins. (**B**) HA-specific antibody titers elicited by the vaccinations were measured by ELISA using the E. coli-expressed HA proteins as coating antigens. Data are the mean of each cohort (*N* = 5), and the error bars indicate standard deviation (SD).

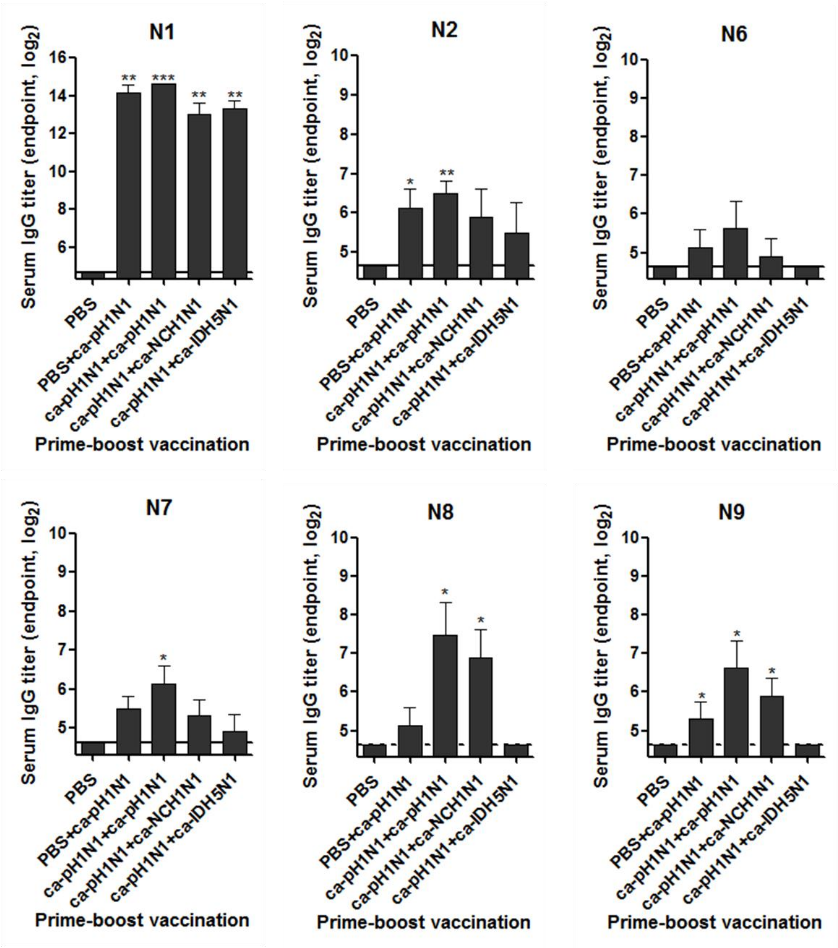

Supplementary Figure 2. NA-specific cross-reactive antibody responses elicited by vaccination. To examine the binding of vaccination-induced sera IgG antibodies to influenza NA proteins, six different recombinant NA proteins expressed in insect cells or human cells were used as coating antigens in ELISA. The NA proteins are derived from A/California/04/2009 (H1N1), A/Aichi/2/1968 (H3N2), A/mallard/Ohio/657/2002 (H4N6), A/Netherlands/219/2003 (H7N7), A/duck/Guangdong/E1/2012 (H10N8), and A/Anhui/1/2013 (H7N9). The antibody titers were expressed as reciprocal serum dilution that yielded OD_{450} value greater than the mean + 2 SD of the PBS control group. Data are the mean of each cohort ($N = 5$), and the error bars indicate SD. ***, $P < 0.001$; **, $P < 0.01$; *, $P < 0.05$ when comparing the antibody titers between the vaccination group and PBS group.

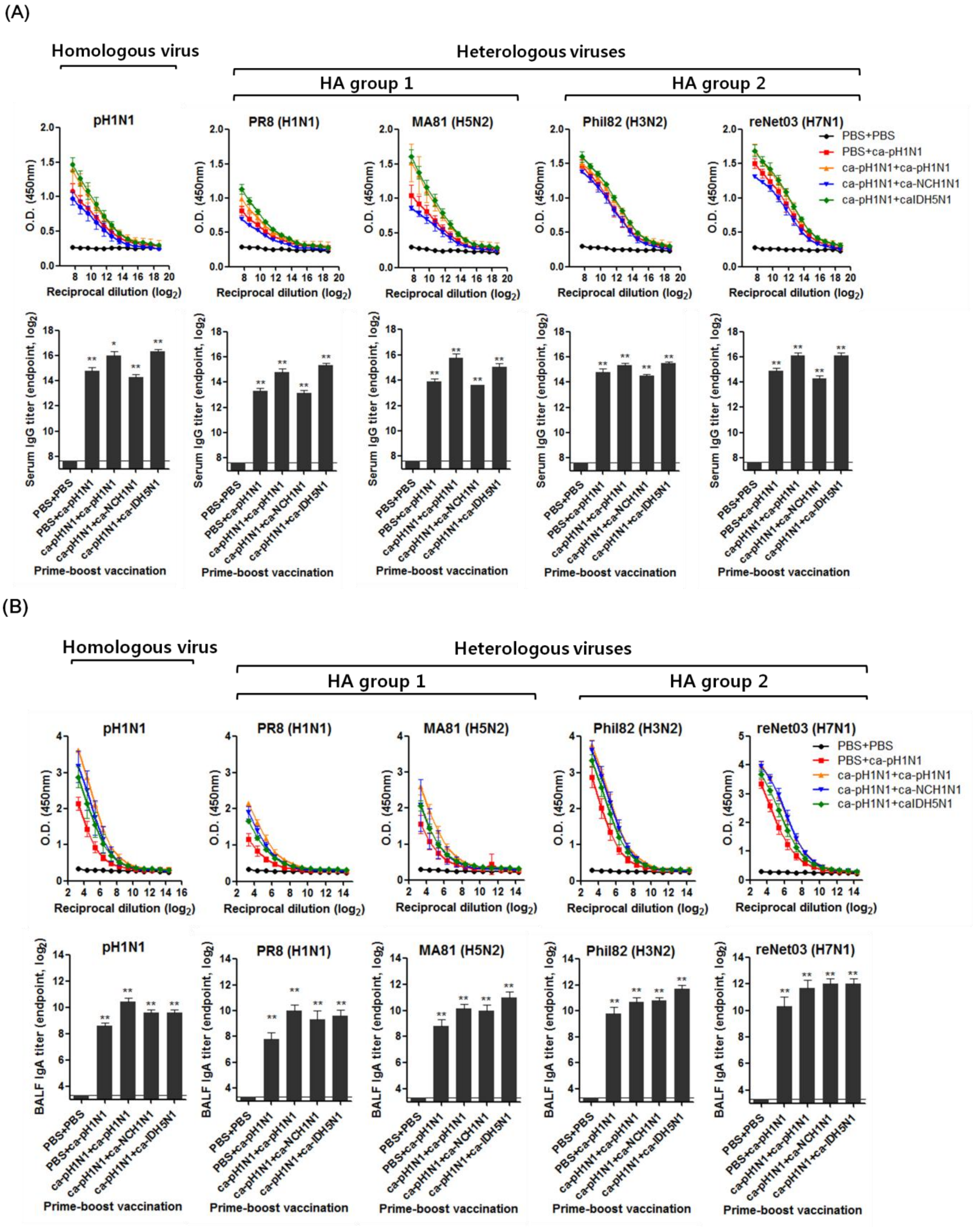

Supplementary Figure 3. Anti-influenza antibodies induced by vaccination. (**A**,**B**) Vaccination-induced antibody titers to purified whole influenza virus virions. Sera and BALF were harvested from mice at eight weeks post-vaccination, and ELISA was performed to estimate influenza-specific sera IgG antibodies (**A**) and BALF IgA antibodies (**B**) against homologous pH1N1 and four heterologous influenza viruses. Antibody titers were expressed as

reciprocal dilution that yielded OD_{450} value greater than the mean + 2 SD of the PBS control group. Data are the mean of each cohort (N = 5), and the error bars indicate SD. ***, $P < 0.001$; **, $P < 0.01$; *, $P < 0.05$ when comparing the antibody titers between the vaccination group and PBS group.

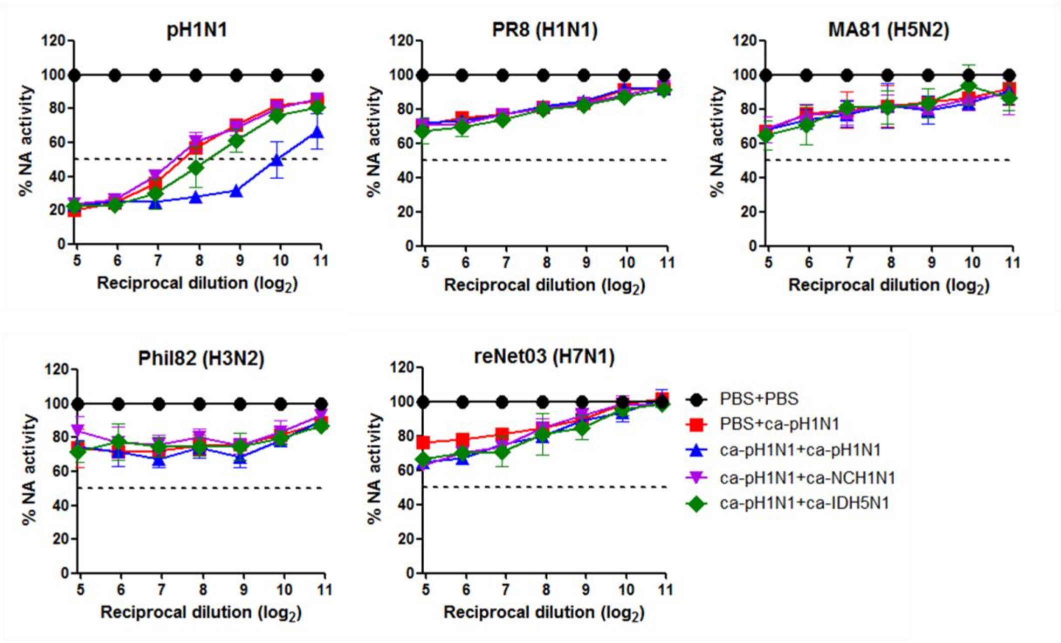

Supplementary Figure 4. NA-inhibition activity of vaccination-induced antibodies. Two-fold serial dilutions of sera from vaccinated mice (N = 5) were incubated with predetermined titer of each virus, and the mixtures were subjected to enzyme-linked lectin assay to measure the NA activity. Data are the mean of each cohort, and the error bars indicate SD.

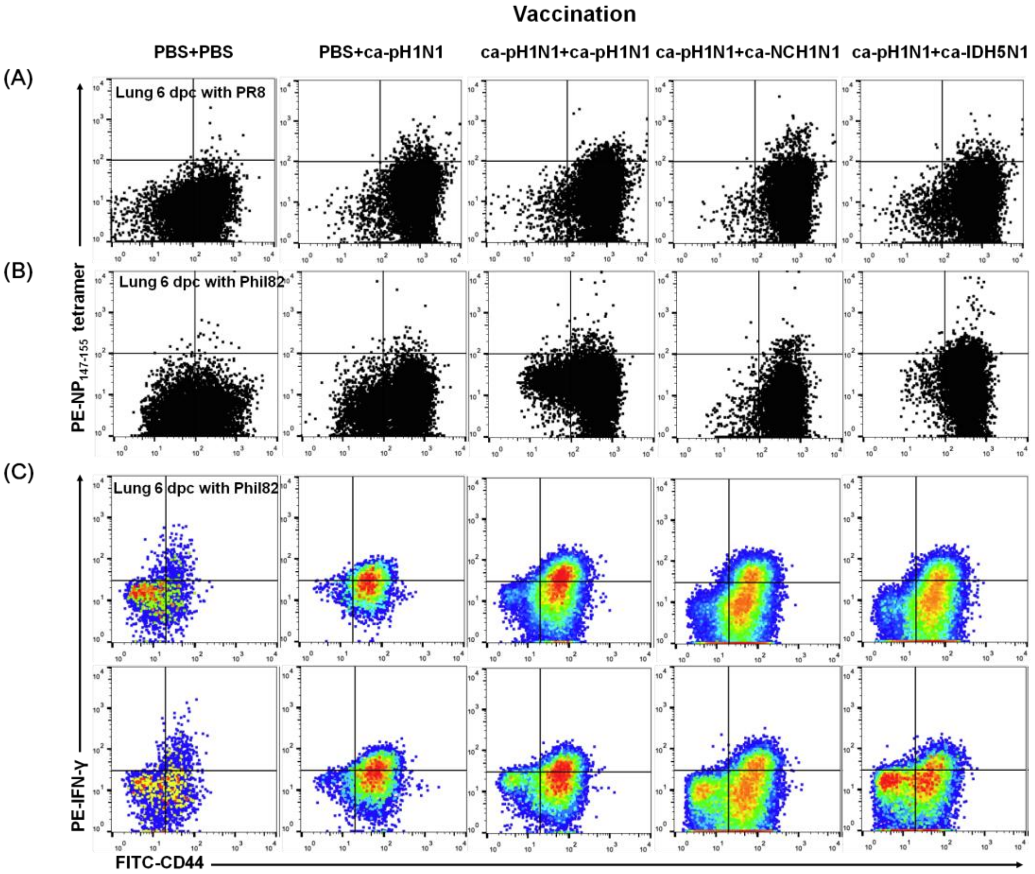

Supplementary Figure 5. Recall responses of CD8+ CTLs upon heterologous challenge. (**A,B**) Flow cytometry analysis of NP$_{147\text{-}155}$+ CD8+ T cells in the lung of vaccinated mice upon challenge with 10 MLD50 of PR8 (H1N1) (**A**) or Phil82 (H3N2) (**B**). Mice lung cells were stained with anti-CD8 mAb, anti-CD44 mAb, and MHC class-I:NP$_{147\text{-}155}$ tetramers before analysis. Data are shown for one representative out of three (PR8 challenge) or five (Phil82 challenge) mice. (**C**) Flow cytometry analysis of IFN-γ producing CD8+ T cells in the lung of vaccinated mice upon challenge with 10 MLD50 of Phil82 (H3N2). Lung cells were stained with anti-CD8 mAb, anti-CD44 mAb, and anti-IFN-γ mAb before analysis. Data are shown for two representatives out of five mice.

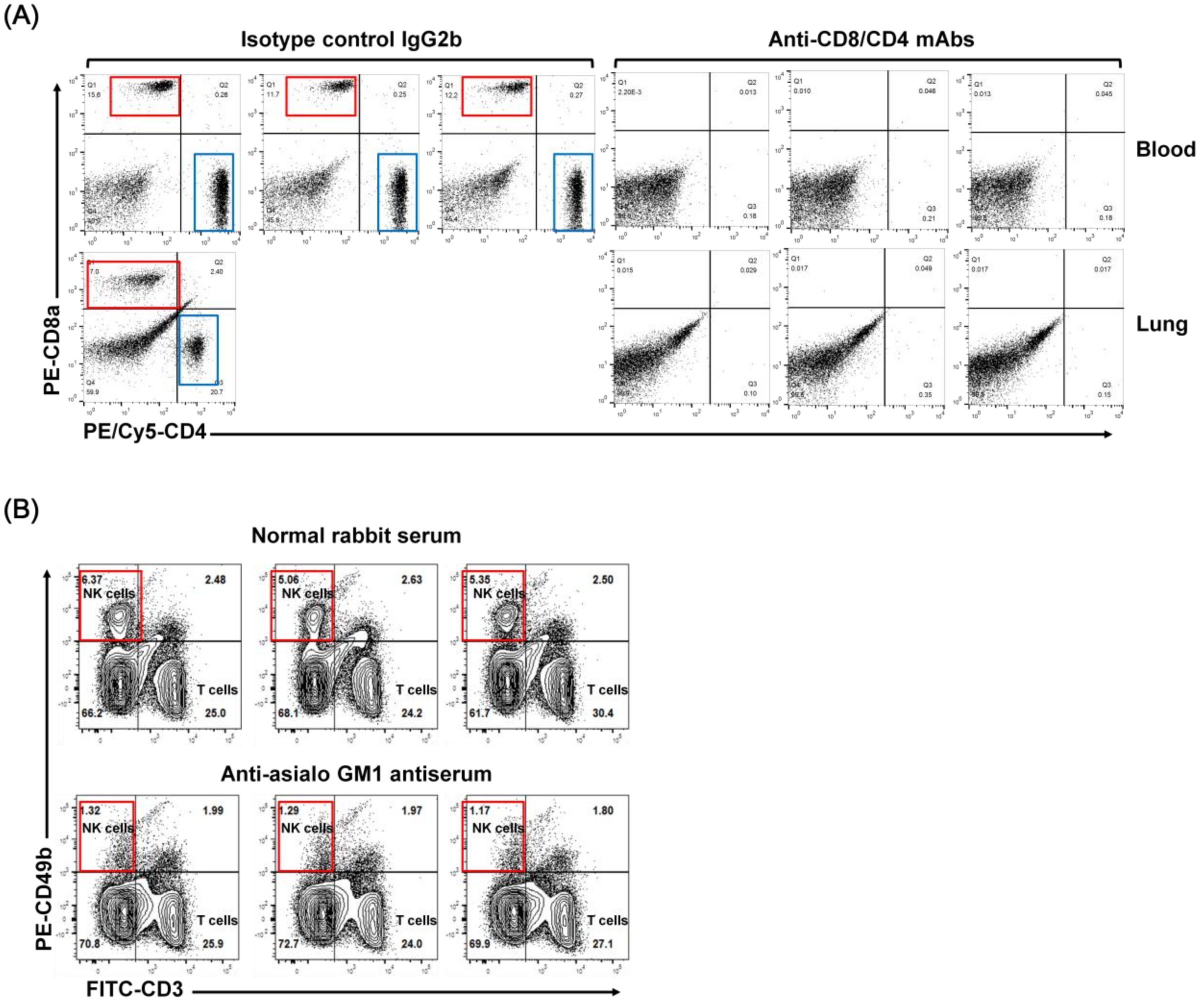

Supplementary Figure 6. *In vivo* depletion of T cells or NK cells by antibody injection. (**A**) For the depletion of CD8+ T cells and CD4+ T cells *in vivo***,** 200 ug of anti-CD8 mAb and anti-CD4 mAb were injected intraperitoneally into mice (*N* = 1 or 3) four times with an interval of two days. Control mice were given isotype control IgG2b antibodies. To confirm the depletion, blood and lungs were taken from the mice 24 h after the last antibody injection and subjected to flow cytometry. The cells were stained with anti-CD8 mAb and anti-CD4 mAb, both of which are different clones to the depleting antibodies. The flow cytomery analysis confirmed the depletion of >99.5 % and >98.3 % of each T cells from the whole lymphocytes in blood and lungs, respectively. (**B**) For the depletion of NK cells, mice (*N* = 3) were intraperitoneally injected with 20µl of anti-asialo GM1 antiserum four times with an interval of two days. Control mice were given normal rabbit serum. Spleens were taken from the mice 24 h after the last injection to confirm the depletion by flow cytometry. The spleen cells were stained with anti-CD3 mAb and CD49b mAb and subjected to flow cytometry analysis to determine the frequencies of NK cells (CD3- CD49b+) and CD3+ T cells.
